# Absolute scaling of small- and wide-angle X-ray scattering images recorded with short duration X-ray pulses on a large area fiber-taper X-ray detector

**DOI:** 10.1107/S1600577526001451

**Published:** 2026-03-11

**Authors:** Hyun Sun Cho, Friedrich Schotte, Robert Henning, Philip A. Anfinrud

**Affiliations:** a Laboratory of Chemical Physics, NIDDK, NIH, Bethesda, MD, USA; b Center for Advanced Radiation Sources University of Chicago Chicago IL USA; RIKEN SPring-8 Center, Japan

**Keywords:** SAXS/WAXS scattering, partially transmissive beamstop, X-ray detector uniformity correction

## Abstract

We have developed an X-ray scattering setup capable of capturing time-resolved small-angle X-ray scattering (SAXS) and wide-angle X-ray scattering (WAXS) images spanning *q* = 0.02 to over 5.2 Å^−1^ on a single, large area Rayonix MX340-HS detector with time resolution as short as 120 ps. A key feature of this setup is a 0.51 mm-diameter partially transmissive beamstop that enables non-invasive, image-by-image recording of direct beam position and intensity during acquisition. This beamstop attenuates 12 keV undulator radiation by approximately eight orders of magnitude while suppressing off-axis second harmonic radiation. Continuous monitoring of the direct beam position facilitates long-term beam alignment and allows datasets acquired at different times to be placed on a common absolute scale prior to differencing. The accuracy of the difference scattering curves is ultimately limited by the performance of the large area, fiber-taper X-ray detector used in this study. To address accuracy issues, we present a detailed statistical characterization of the detector readout noise and responsivity and introduce a variance-per-count statistic that enables identification of zinger-free averages, generation of precise uniformity corrections, and statistically weighted conversion of two-dimensional scattering images into near shot-noise-limited one-dimensional scattering curves. The detector point-spread function and its effects on resolution and scaling are examined using scattering data from a fused silica plate and from apoferritin solution in a capillary. The ability to acquire high accuracy, high precision scattering curves over a broad range of *q* and temperatures provides a robust foundation for time-resolved studies of biomolecular structure and dynamics in solution.

## Abstract 2

An X-ray scattering setup designed to capture small- and wide-angle X-ray scattering on a single, large area detector features a partially transmissive beamstop that facilitates non-invasive recording of X-ray beam position and intensity during acquisition of X-ray scattering images. The integrated transmitted intensity allows different datasets to be put on the same absolute scale and thereby achieve accurate differencing.

## 1. Introduction

Synchrotron beamlines specializing in X-ray scattering studies of biomolecules typically focus on the small-angle X-ray scattering (SAXS) region where background scatter is low, and often employ long flight paths through vacuum between sample and detector (Koch, 2010 ▸; Jeffries *et al.*, 2021 ▸). While the SAXS region provides useful low resolution information such as the size and shape of a scattering particle, the wide-angle X-ray scattering (WAXS) region can provide complimentary insights at higher resolution, such as the secondary structure of biomolecules (Svergun & Koch, 2002 ▸; Makowski, 2010 ▸). Positioning a large area detector close to the sample gains access to the WAXS region, while shrinking the dimension of the beamstop preserves access to the SAXS region. With a relatively short helium-purged X-ray path between sample and beamstop, the benefits of vacuum diminish, with an ‘open’ environment around the sample location providing far greater flexibility for visualizing and positioning the sample, controlling its temperature, and, when pursuing time-resolved studies, triggering structural changes via a variety of approaches including stop-flow mixing or laser excitation (Cho *et al.*, 2021 ▸; Kirby & Cowieson, 2014 ▸).

Here, we describe an X-ray scattering setup that spans *q* = 0.02 to over 5.2 Å^−1^ in reciprocal space, which corresponds to real space limits of approximately 1.2 to 300 Å. Given our aim to ‘leave no peak behind’, the high-resolution limit was set to be comparable with the shortest molecular bond lengths exhibited in heavy atoms. In addition to broad *q* coverage, many scattering studies critically depend on the ability to place datasets acquired at different times on a common absolute intensity scale, particularly when extracting small difference signals. This setup features a small, partially transmissive beamstop that enables precise, non-invasive measurement of the incident X-ray beam position and intensity for each image acquired. This information is used to not only properly scale images but also to maintain long-term beam alignment.

A central objective of this work is to enable accurate absolute scaling of SAXS/WAXS datasets, particularly for difference measurements between data acquired at different times with varying incident X-ray intensity. The partially transmissive beamstop provides the internal normalization required to place such datasets on a common absolute scale; however, achieving this level of scaling precision exposes limitations in detector performance that must be quantitatively characterized and corrected.

As will be described, all images acquired by the large area X-ray detector used in this setup suffer from zingers, where pixels or clusters of pixels light up relative to their neighbors due to cosmic rays or natural radioactive decay (see Section 2.4). Though statistically rare (∼10^−13^ s^−1^ pixel^−1^), they can be intense and corrupt the image. When the detector statistics are known, zingers contaminating a single image can be flagged using objective statistical criteria and corrected. This capability is particularly crucial in time-resolved studies where images can differ significantly from one image to the next, which can cause conventional zinger-suppression schemes that compare back-to-back images to fail. To that end, we perform a detailed statistical characterization of a large area X-ray detector and introduce a variance per count statistic, **VPC**, that facilitates the flagging of zingers in single images and ensures proper weighting of individual pixels when performing weighted averages.

When characterizing the detector point-spread-function, **PSF**, we discovered a weak, broad pedestal that leads to scaling errors near the boundaries between CCD modules and, more importantly, near the edge of the beamstop. Here, we describe methods developed to compensate for these detector scaling errors and describe approaches to properly subtract Compton scattering. For illustrative purposes, we characterize scattering from a fused silica plate and from apoferritin solution flowing in a capillary.

The ability to acquire high-precision scattering data over a large range of *q* with short duration X-ray pulses and an open sample environment enables acquisition of time-resolved, high-resolution scattering data from a variety of samples over a broad range of temperatures.

## 2. Experimental

**Figure 1:**
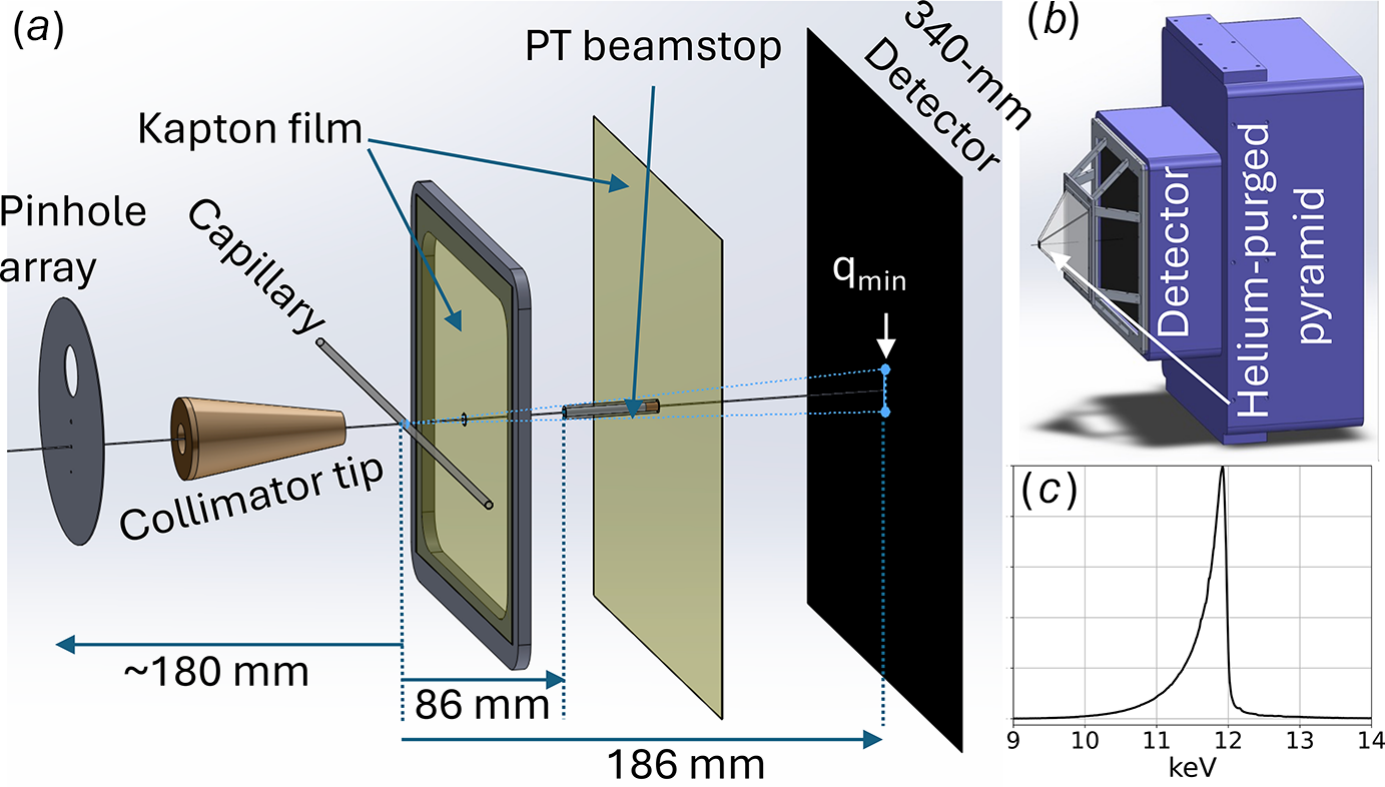
X-ray beam path. (*a*) Scaled drawings of components along the X-ray beam path: pinhole array, collimator tip, sample-filled capillary, 12 µm thick polyimide film with 500 µm pinhole, partially transmissive beamstop bonded to a 100 µm thick polyimide film, and X-ray detector. The X-ray beam passes through a 75 µm × 150 µm (V × H) pinhole cut in a 50 µm thick tungsten wafer, through a brass collimator tip with a 150 µm aperture, and then through the sample. The 2.5 mm gap between the collimator tip and the sample is drawn to scale, but the 186 mm distance between the sample and detector plane is not. A small portion of the X-ray beam leaks through the 3.7 mm long, 0.51 mm OD partially transmissive PT beamstop and impinges on the detector. The shadow cast on the detector by the beamstop defines *q*_min_, which is just under 0.02 Å^−1^ with 12 keV photons. (*b*) Scaled drawing of the Rayonix MX340-HS detector with a helium-purged pyramid mounted in front. The ribbed structure supporting the pyramid is designed to position the apex and base polyimide films 2.5 mm and 90 mm downstream from the capillary, respectively. The position of the beamstop relative to the detector is controlled by manual X and Y micrometres attached to the ribbed structure (not shown). (*c*) The asymmetric pink-beam spectrum, recorded with a Si 111 channel-cut monochromator, is peaked near 12 keV and has a FWHM of approximately 3%.

The infrastructure needed to pursue temperature-dependent, time-resolved X-ray scattering studies of biomolecules was developed on the BioCARS 14IDB time-resolved beamline at the Advanced Photon Source (APS) (Cho *et al.*, 2010 ▸; Graber *et al.*, 2011 ▸; Henning *et al.*, 2024 ▸; Cho *et al.*, 2018 ▸). This beamline employs a set of choppers and shutters capable of isolating a single X-ray pulse or burst of X-ray pulses produced by a pair of short period undulators (U23 and U27) at a repetition rate up to 1 kHz. The undulator gaps were set to produce 12 keV photons and the upstream white-beam slits were narrowed to transmit a 3% (FWHM) slice of the ‘pink’ beam [see Fig. 1 ▸(*c*)]. Primary and secondary KB mirror pairs control the X-ray spot size on the sample. When the APS is operated in hybrid mode, the shutter system could isolate either the super bunch, which consisted of eight septuplets (56 pulses) spanning 500 ns (86 mA), or a single bunch of approximately 120 ps duration (16 mA) (Graber *et al.*, 2011 ▸). When operated in 24-bunch mode, the shutter system is capable of isolating a single pulse (4.25 mA) from the pulse train, or a train of *N* pulses separated by 153 ns. The hybrid super bunch delivered about 125 µJ per burst (6.5 × 10^10^ photons) to a 40 µm × 40 µm spot on the sample, while the hybrid single bunch delivered approximately one-fifth the energy. Note that the beam was focused rather loosely to limit X-ray absorption-induced heating of the sample by the hybrid superbunch. This high flux ‘pink’ beam enables fast time-resolved scattering studies of samples via the pump–probe method with time resolution down to 120 ps (Cho *et al.*, 2010 ▸; Cho *et al.*, 2016 ▸). Since the X-ray beam is not monochromatic, the scattering pattern recorded with this source will be affected, but in a modest and predictable fashion.

The X-ray beam path beyond the focusing optics is illustrated in Fig. 1 ▸. The beam first passes through a 75 µm × 150 µm (V × H) elliptical pinhole (one of several pinhole options in the pinhole array), travels ∼180 mm along a helium-purged path through the 150 µm exit aperture on the brass collimator tip, passes through the sample, and proceeds just under 90 mm to the 510 µm diameter partially transmissive beamstop. Photons scattered from the sample are imaged with a Rayonix MX340-HS detector positioned 186 mm downstream from the sample. With this geometry, the range of *q* from beamstop edge to corner of the detector spans from 0.02 to over 5.2 Å^−1^. The sample can be either a fluid-filled, thin-wall, fused silica capillary or a solid rectangular plate. The sample is supported on a fast linear motor translation stage capable of up to 5*g* acceleration along the long axis of the sample (not shown). Forty X-ray shots were integrated on the detector before readout with the sample translated 0.5 mm between each shot, which minimized the risk of X-ray induced changes to the scattering pattern.

### 2.1. X-ray pulse energy monitor

**Figure 2:**
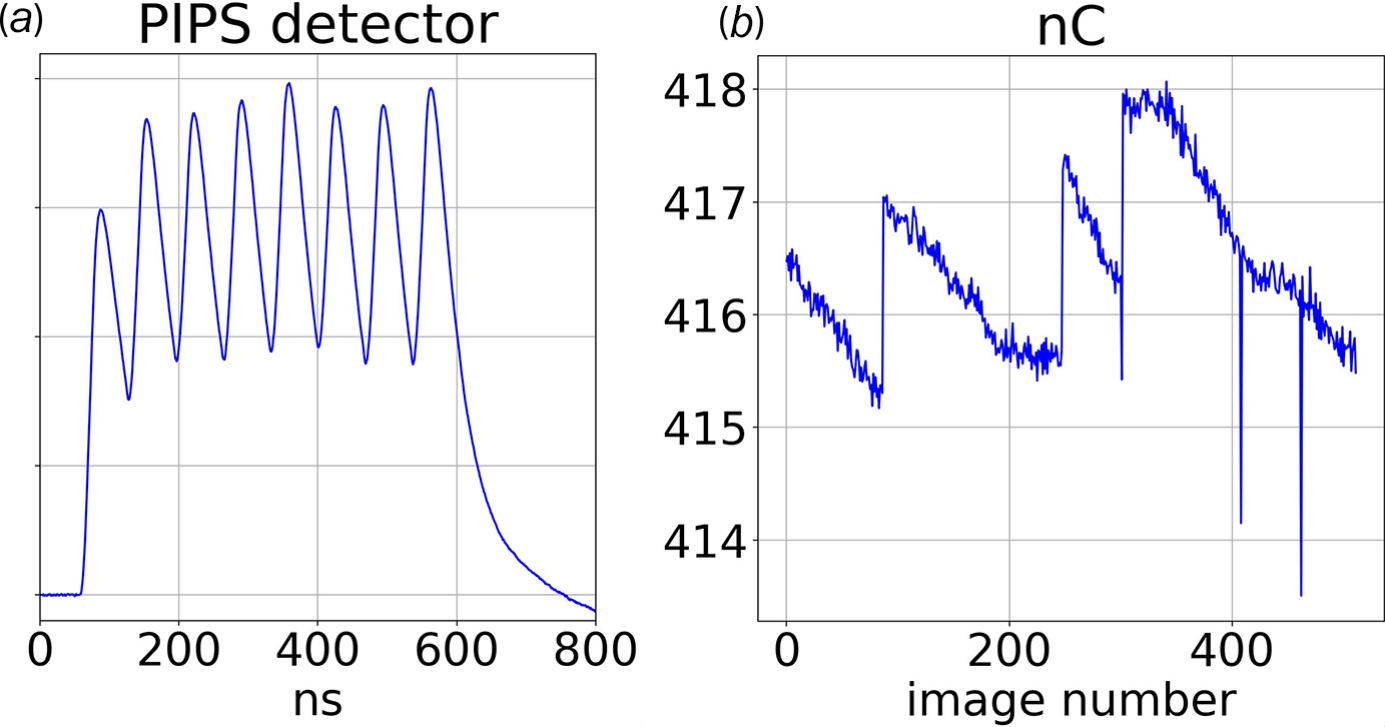
PIPS detector. (*a*) Trace of PIPS detector signal generated from the hybrid superbunch, which consists of eight septuplets and spans 500 ns. The time resolution of the PIPS detector is insufficient to resolve the septuplets, which are spaced by 2.84 ns. The integral of this pulse train corresponds to approximately 2.6 nC charge, which in turn corresponds to 6.5 × 10^10^ 12 keV photons. (*b*) Integrated charge recorded for 512 consecutive images with each representing the integral of 160 superbunches. The intensity decays at a characteristic rate with periodic top-up boosting the integrated bunch charge.

The pulse energy of the pink beam transmitted through the BioCARS shutter system is non-invasively monitored by an annular passivated implanted planar silicon (PIPS) detector (Canberra ANFD300-20-300RM) positioned near the last beryllium window along the vacuum beam path and just upstream of the helium-purged, secondary KB focusing mirrors enclosure (not shown in Fig. 1 ▸). Whereas the direct beam passes through the central hole in the PIPS detector unimpeded, X-ray photons diffracted by the beryllium window impinge on the PIPS detector, which creates hole–electron pairs whose integrated charge is recorded by a LeCroy digital oscilloscope [see Fig. 2 ▸(*a*)]. The PIPS signal is capacitively coupled to the oscilloscope through a biased ‘T’ (9 V) and 50 Ω terminated. The resulting bipolar signal is integrated up to the zero crossing and converted to nanocoulombs. A single APS hybrid mode superbunch, which accounts for 86 mA of the 102 mA operating current, generates approximately 2.6 nC charge on the PIPS detector. Assuming 3.62 eV per hole–electron pair, the number of 12 keV photons absorbed by the PIPS detector per superbunch is estimated to be about 4.9 × 10^6^.

The average energy of the X-ray pulse or pulse train delivered to the sample is determined using a laser joule meter with the hybrid superbunch delivering approximately 125 µJ to the sample. This energy corresponds to approximately 6.5 × 10^10^ 12 keV photons and provides the calibration factor needed to convert nC to X-ray photons, *i.e.* 2.5 × 10^10^ 12 keV photons incident on the sample per nC charge recorded by the PIPS detector. The integrated PIPS signal for 512 consecutive images, shown in Fig. 2 ▸(*b*), with each value representing the sum of 160 shots and tracks time-dependent changes in the incident X-ray flux due to the gradual loss of bunch charge and top-up replacement of the lost charge. Note that top-up of either single bunch or super bunch momentarily disrupts the electron bunch position in the synchrotron ring and can transiently reduce X-ray transmission through the slits and pinholes along the beam path from source to collimator tip [see three negative-going excursions in Fig. 2 ▸(*b*)].

### 2.2. Helium-purged pyramid

It is common practice to position the beamstop on or near the X-ray detector. However, when the flight path from sample to detector is helium purged, not vacuum, the beamstop should be located as far as possible from the detector to shorten the helium scattering path. The beamstop should be as small as possible to retain access to small-angle scattering but must be large enough compared with the X-ray beam size to prevent spillage of the direct beam. A good compromise places the beamstop near the midpoint between the sample and the X-ray detector, as shown in Fig. 1 ▸. Given the 340 mm square dimension of the detector and the relative position and dimension of the beamstop, the range of *q* accessible with this layout spans from 0.02 to over 5 Å^−1^.

The helium-purged enclosure is a 45° angle pyramid assembled from four identical panels of polycarbonate and glued to an aluminium support structure. The base of the pyramid is sealed with a 100 µm thick polyimide film and the partially transmissive beamstop is bonded to the film with ep­oxy. The aluminium support structure is screwed to the front face of the X-ray detector with micrometre adjustments to precisely center the beamstop on the X-ray beam. When the apex of the pyramid was sealed with a 500 nm thin silicon nitride membrane (Norcada), ongoing X-ray exposure contributed to increasing SAXS scattering from the membrane, presumably due to oxidation of the surface layer by X-ray generated ozone. To avoid this problem, the silicon nitride membrane was replaced with a 0.3 mm thin square steel frame on which a 12 µm thin film of polyimide with a central 0.5 mm pinhole was bonded. Since the direct beam does not pass through the film, it does not contribute any additional scattering, and a modest helium purging rate is sufficient to prevent air from entering the enclosure through the pinhole.

### 2.3. Partially transmissive beamstop

Semi-transparent/active beamstops incorporating photodiodes have been used for simultaneous measurement of the transmitted beam intensity in SAXS/USAXS instruments (Krosigk *et al.*, 2001 ▸). Our implementation differs in that the attenuated direct beam is recorded on the same large area detector as the scattering signal, enabling per image intensity normalization as well as beam position tracking. Moreover, the beamstop is designed to address the problem of harmonic contamination and tolerate exposure to high flux, short-duration X-ray pulses without melting or ablating.

**Figure 3:**
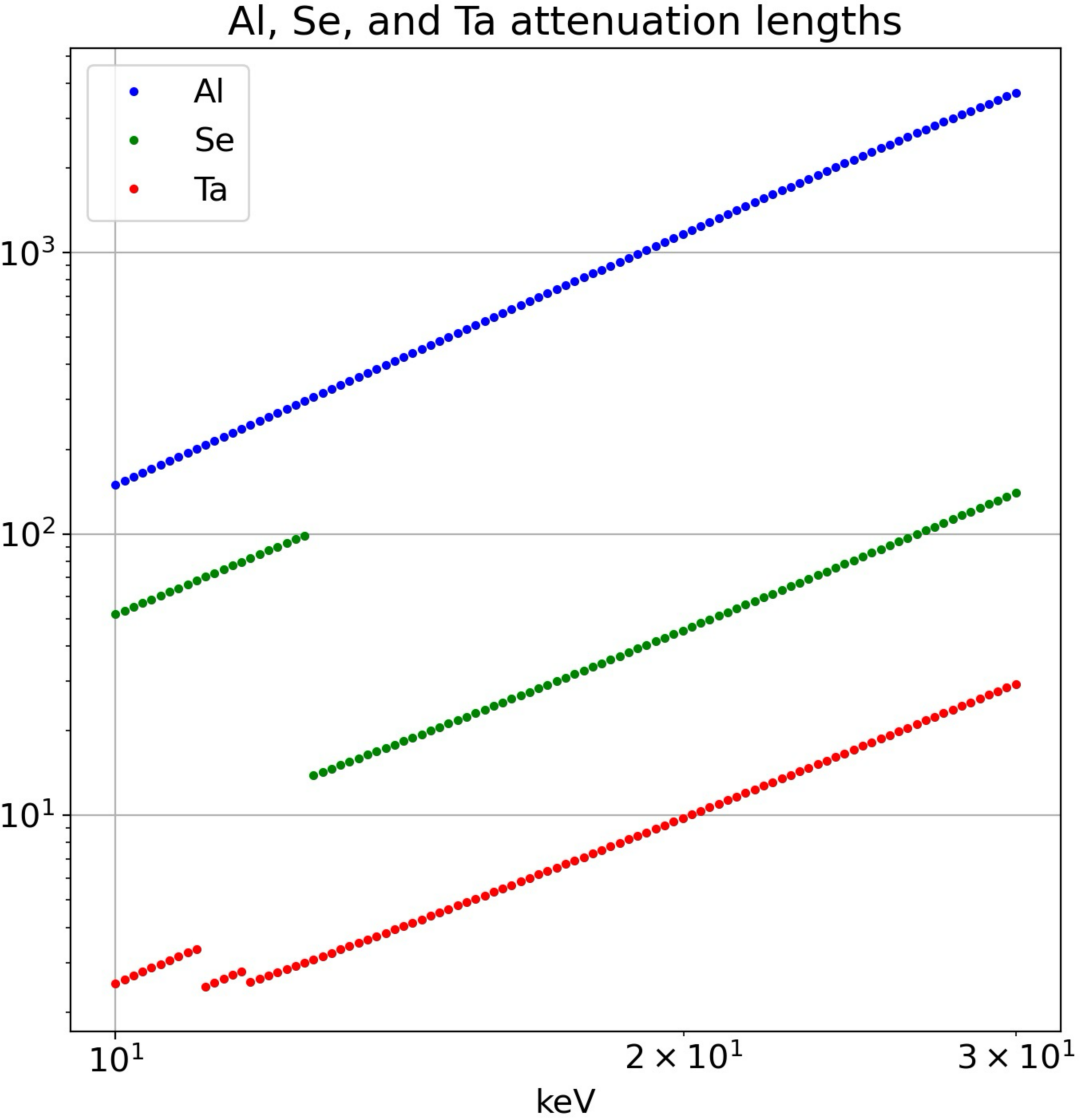
X-ray energy-dependent attenuation lengths for materials used in the partially transmissive beamstop.

Whereas most of the pink beam flux generated from a short-period undulator arises from the on-axis fundamental, the beam is contaminated by far weaker off-axis second harmonic radiation. Hence, design considerations for the partially transmissive beamstop include the following: (1) the partially transmissive beamstop must provide approximately eight orders of magnitude attenuation and tolerate intense bursts of X-rays without melting or ablating; (2) its diameter must be small enough to access SAXS scattering down to *q* = 0.02 Å^−1^ at a sample-to-detector distance sufficiently close to access WAXS scattering beyond 5 Å^−1^; (3) it must not only attenuate the on-axis 12 keV fundamental but also suppress the weak, off-axis second harmonic that contaminates the undulator radiation. To achieve these objectives, we fabricated a partially transmissive beamstop using three different materials. See Table 1 ▸ for their properties and Fig. 3 ▸ for their energy-dependent attenuation length. A 0.51 mm outer-diameter (OD), 0.21 mm inner-diameter (ID) tantalum tube was acquired from Goodfellow Corp., bored to 0.34 mm, and cut to a length of 3.7 mm. A high purity (99.999%) 5 mm diameter aluminium rod was acquired from Goodfellow Corp. and turned on a lathe to produce a long 0.32 mm OD whisker. The aluminium whisker was inserted into the tantalum sleeve with its flat end positioned 0.5 mm from its end and the remaining volume was packed with selenium powder. The sleeve was heated above the melting point of selenium, which flowed to occupy the empty space and upon cooling bonded the aluminium whisker to the tantalum sleeve. The excess aluminium was cut off and the sleeve polished flat on both ends. The predicted transmittance of 12 keV photons through 3.2 mm of aluminium (4 × 10^−6^) and 0.5 mm selenium (2 × 10^−3^) is nearly 10^−8^. For 24 keV photons, the transmittance is estimated to be 2.5 × 10^−4^, which is sufficiently low to suppress the weak second harmonic contamination. Note that this degree of second harmonic suppression requires the polychromatic X-ray spectrum be confined below the threshold where the attenuation length in selenium suddenly drops, *i.e.* below 12.658 keV. Thanks to the very short X-ray attenuation length in the tantalum sleeve, scattering from X-rays passing through the aluminium cannot escape the sleeve. Given a flat aluminium–selenium interface, the transmittance through the beamstop is position independent within a central 100 µm radius circle.

Note that tantalum, a refractory metal, can tolerate a temperature jump 7.5 times larger before melting and 2.4 times larger before ablating compared with aluminium. However, since the heat capacity of aluminium is 1.04 times greater than tantalum (per unit volume) and its 12 keV attenuation length is 97 times longer, aluminium can tolerate 13.3 times greater X-ray flux than tantalum before melting and 42 times greater flux before ablating. For example, when 6.5 × 10^10^ 12 keV photons (125 µJ) in a 40 µm × 40 µm spot penetrate the partially transmissive beamstop, about 15% of the X-ray energy is absorbed in the first 40 µm, which would be expected to elevate the temperature in this (40 µm)^3^ volume of aluminium approximately 130°C. The 12 keV flux required to exceed the threshold for aluminium ablation would be approximately 1 × 10^12^ photons in a 40 µm × 40 µm spot, a flux that can be achieved at X-ray free-electron laser beamlines but not synchrotron beamlines.

Use of partially transmissive beamstops in SAXS studies has been reported elsewhere. For example, Lyngsø & Pederson (2021 ▸) reported a zinc-based partially transmissive beamstop that was developed for SAXS studies on a home source; however, they acknowledged that the precision of the direct-beam intensity measurement is compromised by strong SAXS scattering due to detector ‘bleeding’. More recently, Wu & Li (2024 ▸) reported a dual-thickness semi-transparent aluminium beamstop in which they position the step edge (transition from 1 to 1.6 mm thickness) on the beam center. Detailed analysis of the transmitted 2D intensity profile on either side of the step, combined with prior knowledge of the relative intensities of the harmonics represented in the direct beam, is required to extract the contribution of the fundamental to the direct beam intensity, which represents the contribution needed to properly scale images acquired at different times. In contrast, harmonic contribution to the direct-beam intensity recorded with our aluminium/selenium partially transmissive beamstop is negligible, so determination of the integrated X-ray exposure is accurate and far more straightforward. Moreover, its small size makes it possible to acquire both SAXS and WAXS scattering images on a single detector, whose non-invasive determination of the integrated X-ray intensity ensures accurate scaling of images.

### 2.4. Rayonix MX340-HS detector

The large area (340 mm × 340 mm) Rayonix MX340-HS detector employs a thin phosphor film that converts X-rays to visible photons and a 4 × 4 tiled array of 2.92:1 glass fiber tapers that deliver those photons to 16 CCD modules that are cooled to −80°C, each with 16 ADC channels that are read with 16-bit resolution and a DQE of 0.8 counts per 12 keV photon. The raw pixel data are converted to a 3840 × 3840 gap-free virtual image with an effective pixel size of 89 µm at a frame rate up to 10 Hz using a proprietary algorithm that corrects for spatial distortion and detector nonuniformity. The unprecedented specifications achieved by this detector when introduced was made possible thanks in part to its tiled fiber taper array. However, the fiber tapers introduce a few liabilities that need to be managed to gain the full benefits of this detector (Alkire *et al.*, 2016 ▸; Holton *et al.*, 2012 ▸; Uesugi *et al.*, 2011 ▸). For example, virtual images are created by mapping linear combinations of a variable number of raw pixels to virtual pixels, which affects their statistics. Radioactive decay from trace elements in the fiber tapers as well as cosmic rays interacting with the fiber taper generate so-called zingers, which cause clusters of neighboring pixels to return count values outside the norm and need to be flagged as outliers according to statistical criteria (Barna *et al.*, 1999 ▸). The point-spread-function **PSF** for this detector is specified to be 100 µm; however, we found the **PSF** includes a weak but broad pedestal that leads to edge effects that need to be managed (Holton *et al.*, 2012 ▸; Alkire *et al.*, 2016 ▸).

### 2.5. Image correction factors

We ultimately wish to convert 2D scattering images to *I*(*q*), one-dimensional scattering curves that characterize the sample scattering power as a function of *q* in units of photons per solid angle. This conversion requires numerous image corrections (Pauw, 2014 ▸; Skinner *et al.*, 2012 ▸) including the standard polarization **POL** and geometry **GEO** (Bösecke & Diat, 1997 ▸) corrections. The X-ray path between sample and detector phosphor includes absorbing materials whose angle-dependent losses are accounted for by a filter **FLT** correction. The responsivity of the phosphor used by the Rayonix detector to convert X-rays to visible light is incident angle dependent and therefore requires a phosphor responsivity **PR** correction. X-ray absorbance by the sample is angle-dependent and requires a sample absorbance **SAC** correction. Finally, the Rayonix detector uniformity suffers from scaling errors and requires a uniformity correction **UC**. We denote the composite correction factor **CORR** = **POL**·**GEO**·**FLT**·**PR**·**SAC**·**UC**. Note that we use boldface for quantities that map to virtual pixels on the detector. All corrections depend on ϕ, the scattering angle relative to the X-ray path. Corrections that depend on ψ, the scattering azimuthal angle, include **POL**, **UC** and **SAC** if the shape of the sample is not a plate. For example, if the sample flows through a capillary, the absorbance correction for its cylindrical shape is a function of both ϕ and ψ.

#### 2.5.1. POL: polarization correction

The X-ray scattering intensity distribution from a point source with horizontally polarized X-rays is described by
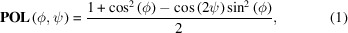
where ϕ is the scattering angle and ψ is the azimuthal angle. Normalizing (dividing) a scattering image by **POL** corrects for this effect and puts virtual pixels at the same *q* on the same scale.

#### 2.5.2. GEO: geometry correction

The X-ray detector reports intensities in terms of counts per pixel. However, what we ultimately desire is the scattering intensity in units of counts per solid angle. Since the X-ray detector is planar, not hemispherical, pixels at high *q* are positioned farther from the source and capture a smaller cross section from the scattered intensity distribution. The intensity in counts per pixel can be readily converted to counts per solid angle by normalizing the image with the geometry correction, which depends only on ϕ according to
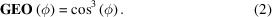


#### 2.5.3. FLT: filter absorbance correction

**Figure 4:**
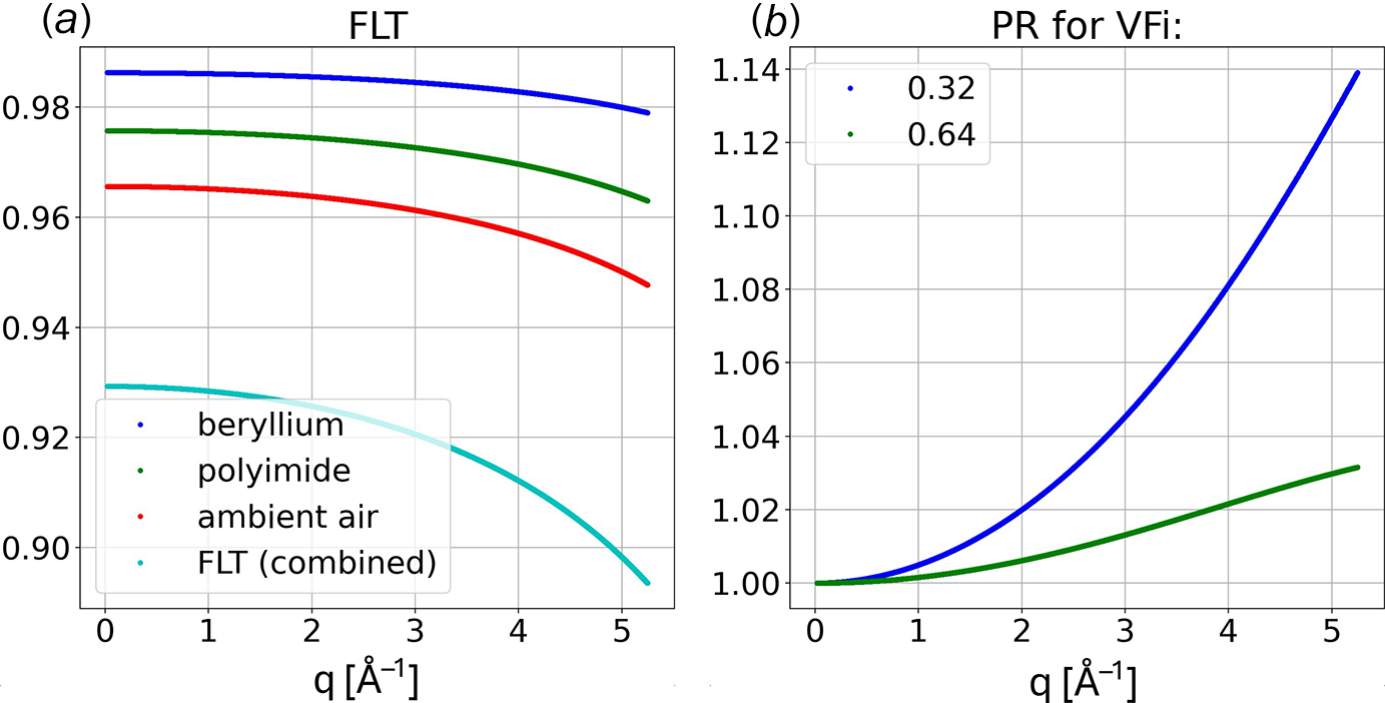
Filter transmittance **FLT** and phosphor responsivity **PR**. (*a*) Transmittance through materials along the path between capillary and detector as a function of *q* with the combined **FLT** corresponding to the product of the transmittances. (*b*) Phosphor responsivity for two assumed phosphor particle volume fractions.

Scattered X-rays are attenuated as they pass through various materials en route to the detector according to their respective attenuation lengths, μ_*i*_. For example, in the experimental layout described in Fig. 1 ▸, scattered X-rays pass through 12 µm polyimide (μ = 4.075 mm) at the apex of the helium-purged pyramid and 100 µm polyimide at the base of the pyramid, 96 mm ambient air (μ ≃ 2750 mm) between the base of the pyramid and the detector, and 200 µm beryllium (μ = 14.49 mm) before encountering the phosphor, which converts X-rays to visible photons. Since μ for 1 atm helium is over 300 m, its contribution can be neglected. The overall transmittance decreases with increasing ϕ according to
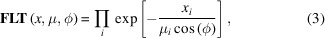
where *x* corresponds to the filter thickness. As shown in Fig. 4 ▸(*a*), absorbance by ambient air makes the largest contribution to the combined **FLT** transmittance.

#### 2.5.4. PR: phosphor responsivity correction

X-rays impinge on a 40 µm thick polymer sheet (paryl­ene_N) in which particles of Gadox phosphor are embedded (Gruner *et al.*, 1993 ▸). The pathlength through the sheet increases with increasing ϕ, which leads to an increased probability of X-ray absorbance and higher phosphor responsivity. The attenuation length for Gadox is rather short, μ_G_ = 8.37 µm, but it is present as fine particles embedded in a polymer with a volume fraction VF. Hence, the phosphor responsivity **PR** might be expected to increase with increasing ϕ according to
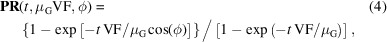
where *T*_0_ = exp(−*t*VF/μ_G_) corresponds to the on-axis phosphor transmittance, which depends on the phosphor sheet thickness *t* and phosphor VF. Neither the shape of the phosphor particles nor their VF in the polymer film is specified by Rayonix. If the phosphor particles are approximately spherical, random close packing places an upper bound on VF of 0.64. To illustrate the VF dependence of **PR**, the chart in Fig. 4 ▸(*b*) includes curves with VF differing by a factor of two: 0.32 and 0.64. The correction at high *q* varies nonlinearly from nearly 14% to 3% over this range. As we will show elsewhere, our best experimental estimate for VF was found to be 0.33.

#### 2.5.5. PT: absorbance correction for a plate scatterer

In this paper, we present scattering data from fused silica and glassy carbon plates for which the sample absorbance correction **SAC** is defined by plate transmittance, **PT**. X-rays passing through a plate at normal incidence suffer absorbance losses according to the material’s attenuation length μ. The absorbance losses experienced by scattered photons depends on the thickness of the plate, the scattering angle, and where in the plate the scattering originated. The angle-dependent transmittance for a plate with thickness *t* and attenuation length μ is given by
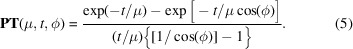
Normalizing the scattering image from a plate by **PT** corrects for absorbance losses in the plate.

#### 2.5.6. UC: uniformity correction

As we will show, Rayonix detector images are recorded with excellent repeatability but suffer from scaling errors that need to be corrected by a uniformity correction **UC** derived from experimental data. Due to the detector point-spread function, generating a uniformity correction that is both precise and accurate over the entire image entails both experimental and theoretical considerations.

### 2.6. Data acquisition protocol

We take advantage of the integrating capability of the Rayonix MX340-HS detector and expose it to numerous X-ray shots before triggering image readout. Each read operation generates heat and elevates the operating temperature of the CCD modules to an extent that depends on the frame rate, which can affect the ADC gain. To maximize detector repeatability, the detector is externally triggered by an FPGA-based timing system at a constant frame rate but only the images wanted are permanently saved to disk. For every image acquired, we process the PIPS signal recorded for all corresponding X-ray pulses and determine their integrated charge, nC, which is proportional to X-ray flux incident on the sample.

Time-resolved SAXS/WAXS studies typically expose the detector to periodic bursts of 40 X-ray shots in pulse trains arriving at nominally 20.6, 41.1 or 246.8 Hz, before readout. For the datasets analyzed in this study, four separate bursts of 40 X-ray shots, each arriving at 246.8 Hz, were integrated on the Rayonix detector before being read at a frame rate of just under 1 Hz with complete datasets consisting of 512 consecutive images (14 GB per dataset). Hence, each image represents the integral of 160 shots for which the incident X-ray flux is approximately 10^13^ photons. This mode of data acquisition was selected to improve the statistics for characterizing the detector performance and to reduce the time required to generate each dataset.

Dark datasets were acquired with three different integration times (D1, D2, D4) with the X-rays blocked. X-ray scattering datasets were acquired from a fused silica plate with two different integration times (FS1, FS2), from a glassy carbon plate (GC), and with no sample in the beam, which characterizes helium scattering from collimator tip to beamstop (H).

## 3. Results and discussion

All X-ray scattering images acquired with the Rayonix detector have a background offset, are contaminated with zingers, include a contribution from helium scattering along the path between collimator tip and beamstop, and include Compton (inelastic) scattering. The scattering intensity distribution recorded on the detector is influenced by X-ray polarization **POL**, sample absorption **SAC**, absorbance by materials along the path between the sample and the detector **FLT**, as well as the scattering-angle-dependent responsivity of the detector phosphor **PR**. Moreover, the incident X-ray flux changes over time, which influences the magnitude of the scattering signal. Here, we describe a framework developed to convert background-corrected, zinger-free, uniformity corrected, 2D scattering images to one-dimensional, Compton-corrected *I*(*q*) scattering curves in units of photons per solid angle. We begin by characterizing M0, which is proportional to the integrated X-ray intensity transmitted through the partially transmissive beamstop, and compare this quantity with nC, which is proportional to the X-ray intensity incident on the sample. We introduce the concept of M0 scaling that facilitates isolation of scattering from the sample. Next, we perform a detailed statistical analysis of dark datasets to characterize zingers, assess the detector readout variance, and identify pixels suffering from leakage current. Based on a statistical characterization of images acquired with the detector illuminated with X-rays scattered from a fused silica plate, we introduce a variance per count **VPC** statistic that facilitates flagging of zingers. When characterizing the detector point spread function, **PSF**, we discovered a broad pedestal that contributes to scaling errors near boundaries between CCD modules and the edge of the partially transmissive beamstop. Scattering images from fused silica and glassy carbon are used to generate a uniformity correction that corrects for pixel-to-pixel scaling errors. Finally, we discuss how **PSF** variation across the fiber taper leads to correctable scaling errors when this detector is used to acquire crystal diffraction images.

### 3.1. M0: X-ray transmission through partially transmissive beamstop

A 2D moments analysis of the direct beam transmitted through the partially transmissive beamstop generates M0, M1X and M1Y, where M0 corresponds to the zeroth moment and quantifies the integrated intensity, and M1X, M1Y correspond to the first moments and quantify offsets from the beam center. The beamline is aligned to ensure that the transmitted X-ray beam is well centered on a single pixel, whose coordinates are denoted (X0, Y0). The beam position, which is determined to a precision of a few micrometres, is monitored and the X-ray beam alignment periodically tweaked as needed to maintain the beam position over time. The PIPS detector signal from the same X-ray pulses that contribute to M0 is recorded and converted to nanocoulombs, nC.

**Figure 5:**
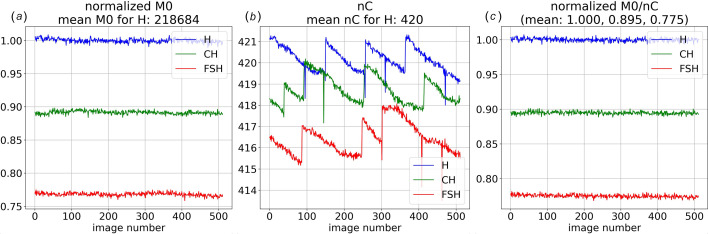
Scale factors for 512 images acquired with the sample retracted (**H**: blue), with an empty capillary inserted into the beam (**CH**: green), and with a 100 µm thick fused silica plate inserted into the beam (**FSH**: red). (*a*) Normalized M0 corresponds to integrated intensity of direct beam transmitted through the beamstop. (*b*) nC corresponds to integrated charge extracted from the upstream PIPS detector. (*c*) M0/nC is normalized relative to **H** and corresponds to transmittance, which for these three curves are 1.00, 0.895 and 0.775. The integrated number of photons incident on the sample was ∼10^13^ photons per image. The integrated number of Rayonix detector counts per image for the **H**, **CH** and **FSH** datasets are approximately 6.7 × 10^8^, 6.3 × 10^9^ and 1.3 × 10^10^, respectively.

Fig. 5 ▸(*a*) charts M0 for three different datasets: **H**, **CH** and **FSH**. **H** was acquired with the sample retracted and quantifies scattering from helium along the X-ray path between the collimator tip and beamstop. **CH** was acquired with a 320 µm OD fused silica capillary inserted into the beam and includes scattering from both capillary **C** and helium **H**. The interior of the capillary was HF etched to thin its walls and reduce its X-ray scattering. **FSH** was acquired with a 100 µm thick fused silica plate and includes scattering from both fused silica **FS** and helium **H**. The magnitude of M0 depends on the incident X-ray intensity, which is proportional to nC, and the X-ray absorbance of the materials through which the beam passes. The M0/nC ratio corrects for time-dependent changes in the X-ray flux and can be used to put images on the same absolute scale with high precision. For example, the standard deviation of the blue curve charted in Fig. 5 ▸(*c*) is 0.0025, which is very close to the 0.0021 shot-noise limit expected assuming an M0 average of 218684 photons per image. The corresponding image-to-image statistical fluctuations for nC is approximately five times smaller, which therefore affords high precision normalization.

From the **FSH** data in Fig. 5 ▸(*c*), the averaged X-ray transmittance *T* through the 100 µm thick fused silica plate was found to be 0.775. Given *T* = exp(−*t*/μ), where *t* is the thickness and μ is the attenuation length of fused silica, we can experimentally determine the plate thickness. Assuming a density of 2.2 for fused silica, its attenuation length at 11.648 keV (center of mass of the ‘pink’ beam spectrum) is predicted to be 396 µm, from which we can estimate the plate thickness: *t* = −ln(0.775)×396 = 101 µm, which is very close to its specified thickness. Similarly, given the transmittance through the HF-etched capillary of 0.895, we can estimate its combined wall thickness (entrance and exit walls) to be 44 µm. Given its OD and combined wall thickness, the capillary ID was experimentally found to be 276 µm.

The ability to record M0 with high precision, as demonstrated here, is not only used to accurately determine the absorbance of samples inserted into the X-ray beam path but is also used to put different datasets on the same absolute scale. Though the precision of the M0 determination is significantly poorer than that for nC due to shot noise, the average M0 for a dataset containing many images has sufficiently high precision to put datasets acquired at different times on the same absolute scale.

### 3.2. Zingers

Zingers strong enough to saturate the detector are rare and easily detected. Weak zingers, on the other hand, are far more abundant and require accurate statistical criteria to detect and reject them. Since the ability to detect weak zingers is limited by readout noise plus shot noise, zingers are best characterized by analyzing a dark dataset. As we will show, the probability of a zinger affecting a pixel after one second integration is ∼10^−13^. Hence, one might conclude the probability that a single virtual pixel can be affected by zingers in more than one image in a dataset would be negligibly small. However, radioactive decay of trace elements in the fiber taper can generate unstable daughter isotopes that decay further, causing lightning to strike more than once. Analysis of dark datasets found numerous instances where zingers affected the same virtual pixel in two different images of a dataset, but not three. Based on this result, we developed a very simple strategy for determining, from a set of *N* consecutive images, their zinger-free mean and variance: (1) compute a running sum **S** and sum of squares **SS** of counts while retaining the two largest and two smallest count values found in the series; (2) subtract the contribution from the two smallest and two largest values from **S** and **SS**; (3) compute a zinger-free mean, **Cmean** = **S**/(*N* − 4), and variance **Cvar** = **SS**/(*N* − 4) − **Cmean**^2^. Note that omitting the two smallest values as well as the two largest values ensures **Cmean** is not skewed by this procedure.

**Figure 6:**
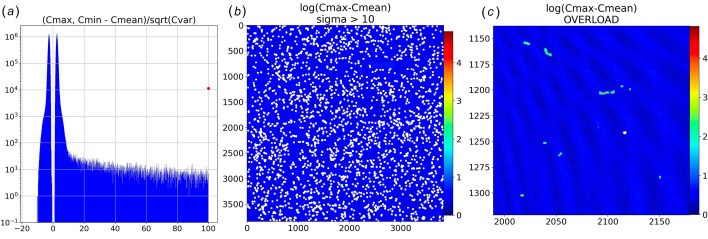
Zingers found in a dark dataset comprising 512 images. (*a*) The sigma histogram exhibits **Cmax**, **Cmin** asymmetry due to zingers; the red dot indicates the number of virtual pixels for which sigma > 100. (*b*) Log of (**Cmax** − **Cmean**) with white dots flagging virtual pixels with sigma > 10. (*c*) Zoomed in image of a region that includes a zinger in which four neighboring pixels overloaded the ADC (flagged by white dots).

Knowing the variance for each pixel is crucial to employ statistical criteria to flag and exclude zingers. For example, in Fig. 6 ▸(*a*), the histogram of the minimum (**Cmin**) and maximum (**Cmax**) count values recorded for every virtual pixel in a Dark dataset of 512 images is charted in terms of **sigma** = (**Cmax**, **Cmin** – **Cmean**)/sqrt(**Cvar**). The distribution corresponding to **Cmin** (negative values) extends to just under 10-**sigma**, which is nearly twice as broad as would be expected if the statistics were normally distributed. If there were no zingers, one would expect the sigma distribution corresponding to **Cmax** (positive values) to be a mirror image of that for **Cmin**. Indeed, the main distribution for **Cmax** appears to be a mirror image, but it includes many virtual pixels that extend well beyond 10-**sigma**. Using a 10-**sigma** threshold as a criterion for flagging zingers [Figs. 6 ▸(*B*)], 21805 zinger pixels were found in 512 images acquired with a total integration time of 572 s, which corresponds to 38.1 zinger pixels per second of detector integration. Thus, the probability of a zinger affecting a single virtual pixel in an image is extremely low: 38.1/3840^4^ ≃ 10^−13^ per second of integration time. A total of 64 virtual pixels found in 13 different zinger clusters overloaded the ADCs, one of which is shown in Fig. 6 ▸(*c*) alongside weaker zingers nearby. As the amplitude of zingers spans a wide dynamic range, it is crucial to know the variance of each virtual pixel to assign a statistically based threshold capable of flagging weak zingers.

### 3.3. Detector offset, leakage current and readout variance

**Figure 7:**
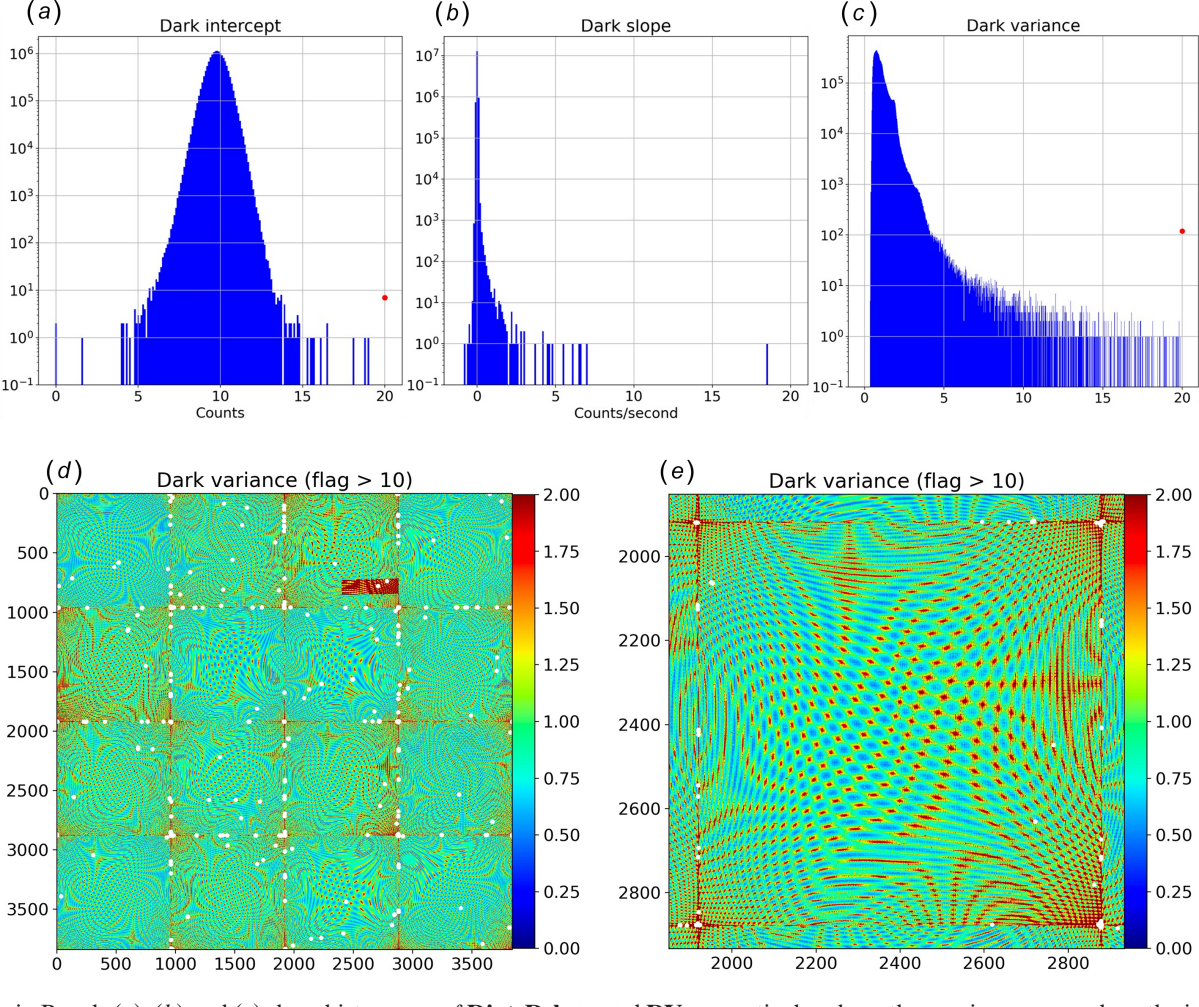
Dark image analysis. Panels (*a*), (*b*) and (*c*) show histograms of **Dint**, **Dslope** and **DV**, respectively, where the *x*-axis corresponds to the indicated quantity. (*d*) Dark variance **DV** image. White dots flag high variance pixels (**DV** > 10). (*e*) The zoomed image corresponds to the CCD module under which the beamstop is placed.

Dark datasets consisting of 512 consecutive images each with X-rays blocked were acquired with three different integration times (D1, D2 and D4) and analyzed to assess the detector background offset, leakage current and readout noise. The zinger-free means for all three datasets were fitted as a function of integration time with a straight line, whose intercept **Dint** corresponds to the background offset [Fig. 7 ▸(*a*)] and whose slope **Dslope** arises from leakage current [Fig. 7 ▸(*b*)]. With the background properly subtracted, the dark image variance **DV** was determined [Fig. 7 ▸(*c*)], which corresponds to the readout noise of the detector after scaling by the effective gain of each virtual pixel.

When initialized, the Rayonix detector measures the background and, when triggered, saves background-subtracted images in an unsigned 16-bit format with a 10-count offset. The zinger-free Dark background offset computed from all three Dark datasets, **Dint**, has a mean of 9.79 counts and a sigma of 0.54 counts but includes about two dozen statistical outliers. Two of those outlier pixels have zero counts and zero variance, which suggests their background determination during the Rayonix initialization step was more than 10 counts below their true mean and were clipped when converting to an unsigned 16-bit integer. Since the background offset generated from the Dark datasets is statistically more precise than that generated during Rayonix initialization, this background offset, not the specified 10 counts, is subtracted from all images acquired in subsequent datasets. This background offset remains valid as long as the Rayonix detector is not reinitialized, after which a new Dark dataset would need to be acquired to minimize error in the background offset.

Pixels with leakage current exhibit a linear increase in background counts with integration time and are identified by a positive slope in the **Dslope** histogram charted in Fig. 7 ▸(*b*). A small number of the nearly 15 million pixels suffer from leakage current with the worst pixel exhibiting leakage of about 18 counts per second. For precise work, the background subtracted from the scattering image should account for the detector frame rate according to
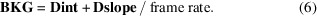
The zinger-free Dark variance **DV**, charted in Fig. 7 ▸(*d*), varies systematically across the virtual image. Note that one of the ADC channels is ill-behaved and was excluded from the virtual pixel data in the histograms. The proprietary algorithm developed by Rayonix to correct for spatial distortion and response nonuniformity generates a gap-free virtual image by weighted mapping of raw pixels to each virtual pixel. By feeding synthetic raw images into this algorithm, we identified which virtual pixels include contributions from which ADC channel and determined how many neighboring raw pixels are mapped onto each virtual pixel. For the Rayonix detector installed on the BioCARS beamline at the APS, that number varies from zero (15356 pixels) to nine (1 pixel), with four being the most common (86%). The virtual pixels for which there is no mapping are all found along the perimeter of the full image and need to be masked. Due to averaging effects, the measured **DV** is expected to scale as the inverse number of raw pixels that contribute to the virtual pixel, and accounts for most of the systematic variation exhibited across the images in Fig. 7 ▸. Note that the 0.75 count peak in the **DV** histogram shown in Fig. 7 ▸(*c*) is similar to the detector DQE, which is specified to be 0.8 for 12 keV photons. Since most virtual pixels represent a weighted average of four real pixels, one would expect the variance of a virtual pixel that maps to a single real pixel to be approximately four times greater, or about 3 counts. However, as shown in Fig. 7 ▸(*c*), **DV** extends well beyond that limit. How might we account for these high variance pixels? Since variance scales as the square of the gain, regions of the detector with poorer photon coupling between the fiber taper and the CCD require higher gain and will exhibit higher variance. For example, fibers along the edges of the fiber tapers are damaged when ground to a square profile for tiling, which reduces the photon coupling efficiency along the perimeter of the fiber tapers and requires higher gain factors to achieve uniform responsivity across the full virtual image. Indeed, as revealed in Figs. 7 ▸(*d*) and 7 ▸(*e*), most but not all high variance pixels are found along boundaries between CCD modules.

### 3.4. PSF: point spread function

**Figure 8:**
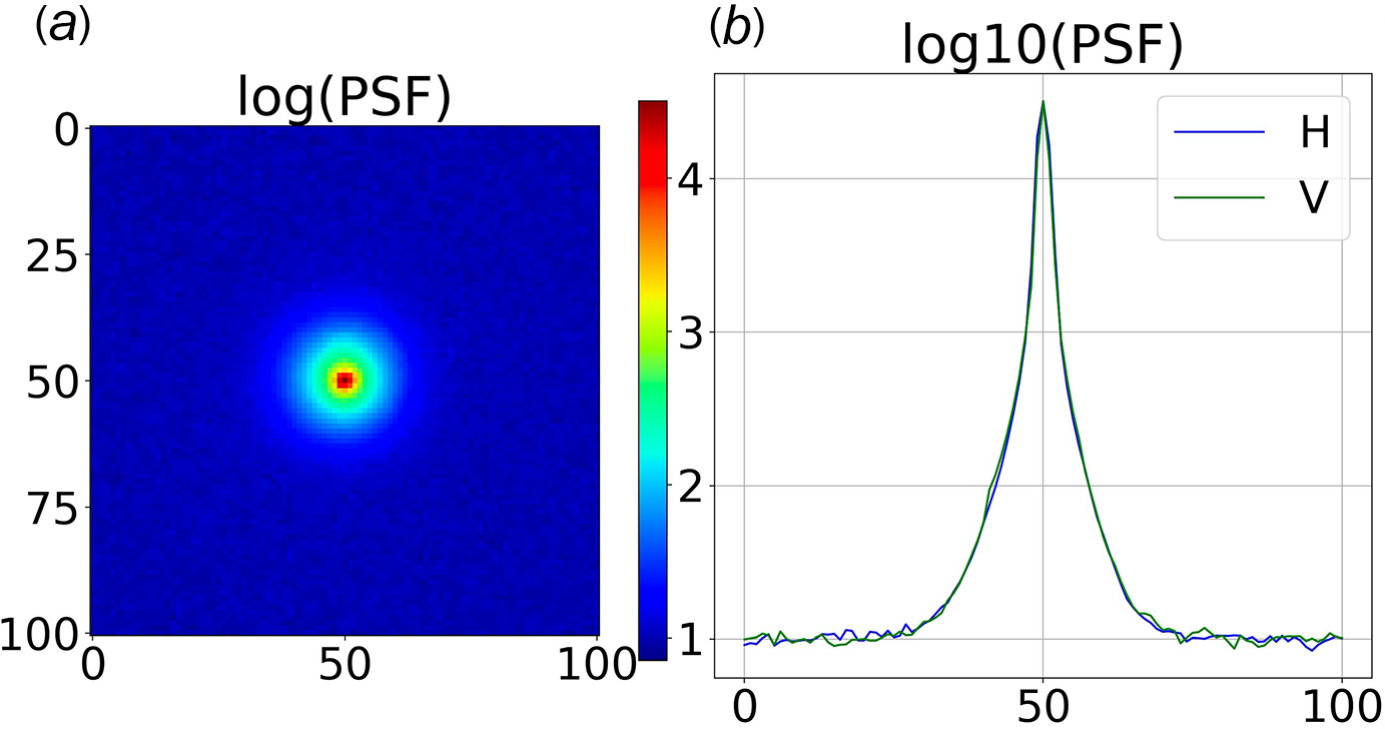
Point spread function **PSF** at the beam center in pixel units. (*a*) Log of highly attenuated direct beam on the Rayonix detector. The intensity distribution is well described by a sum of four Gaussians with the narrow feature (1.94 pixels FWHM) accounting for 58.5% of the photons in the image. The integrated number of counts in this image is approximately 250000. (*b*) Horizontal and vertical slices through the **PSF**. Note the background offset of 10 counts, which translates to 1 on a log_10_ scale.

The 40 µm-thick Gadox phosphor film in front of the tiled glass fiber tapers absorbs X-rays and generates visible photons, a small fraction of which are coupled into the cores of nearby fibers (Gruner *et al.*, 1993 ▸). Photons entering a fiber core at an angle within its numerical aperture can propagate virtually lossless over long distances via total internal reflection. However, since the fiber taper has a 2.92 times smaller core at its exit than its entrance, the numerical aperture at the exit is approximately 2.92 times smaller. Hence, only about 1 in 8.5 (= 2.92^2^) photons remain confined in on-axis fibers, *i.e.* fibers near the center of the fiber taper, with the rest escaping the core and scattering diffusively in the cladding (Coleman, 1985 ▸). The losses are even greater near the corners of the fiber taper: when pulling a fiber bundle to generate a tapered bundle, the pathlength through fibers near the center of the bundle is shortest but increases with distance from center due to the serpentine path taken. Due to conservation of volume, a longer serpentine path implies a smaller diameter core, which exacerbates the losses. Though the cladding is doped with an absorber to minimize transmission of photons that escape the core, a small fraction of the escaped photons are detected, whose spatial profile reflects the diffusive nature of scattering in the cladding. Log charts of a highly attenuated direct beam are shown in Fig. 8 ▸, which is well described by a sum of four Gaussians with FWHM of [1.94, 4.80, 10.0, 18.8] pixels and relative number of integrated counts [0.585, 0.143, 0.174, 0.098]. The narrowest Gaussian corresponds to a FWHM of 173 µm, which is somewhat larger than the Rayonix specification of 100 µm and accounts for 58.5% of the integrated intensity. This ‘spot’-to-pedestal ratio depends on the photon trajectory through the fiber taper and is least favorable in the corners of the CCD modules due to the highly curved serpentine photon path through the fiber taper. Note that for this measurement, the direct beam entered the fiber taper at the beamstop location, which is located approximately 7 mm from a corner and 53 mm (593 pixels) from its center.

The broad pedestal leads to scaling errors near the boundaries of CCD modules and, of greater consequence, in the SAXS region. As we will show, these scaling errors can be quantified and largely corrected.

### 3.5. VPC: variance-per-count statistic

According to photon counting statistics, the variance for *N* photons is equal to *N*. Hence, dividing the variance by the mean produces a variance per count **VPC** statistic that is detector specific and can be used to not only estimate the DQE for the detector but also estimate the variance for each virtual pixel in a single image, which is needed to properly flag zingers.

**Figure 9:**
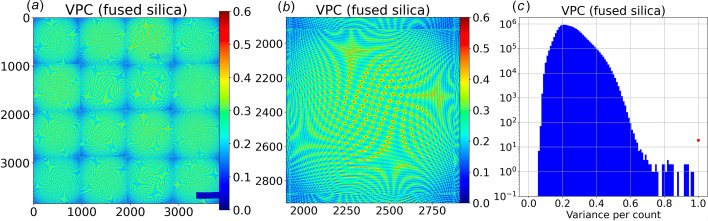
Variance per count **VPC**. (*a*) Full image of **VPC**. The lower aberrant ADC channel shows up more dramatically in the **VPC** statistic while the upper one shows up more dramatically in the **DV** statistic of Fig. 7 ▸(*d*). (*b*) Zoomed-in image of the CCD module above which the beamstop is located (near upper left corner). (*c*) Histogram of **VPC** with pixels from two ill-behaved ADC channels excluded.

The **VPC** statistic was experimentally determined from a set of 512 consecutive fused silica scattering images (Fig. 9 ▸). The algorithm used to generate the zinger-free mean and variance accounted for image-to-image differences in X-ray intensity, which arise due to top-up of the synchrotron fill pattern and would otherwise inflate the measured variance. Briefly, each image in the series is divided by the integral of its background-subtracted counts, IC, before accumulating a running sum and sum of squares. The mean and variance computed from the running sum and sum of squares (after omitting the two largest and smallest values found in the series) are then converted back to counts by multiplying by the mean IC and mean IC squared, respectively.

The experimentally determined variance has contributions from both shot noise and readout noise. We isolate the shot noise contribution and calculate **VPC** according to
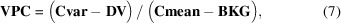
where **DV** is the experimentally determined dark readout variance and **BKG** is the background offset. Hence, **VPC** in combination with **DV** can be used to estimate the statistical uncertainty of every pixel in an image and provides a reliable, statistically based threshold for flagging zingers.

The **VPC** statistic is charted in Fig. 9 ▸ and exhibits systematic variations across the CCD modules that are similar to that observed in **DV** and correlate with the number of raw pixels that map onto the virtual pixels.

A striking qualitative difference between **VPC** and **DV** is a systematic drop in **VPC** that correlates with distance from the center of each of 16 fiber tapers. How might we explain this behavior?

**Figure 10:**
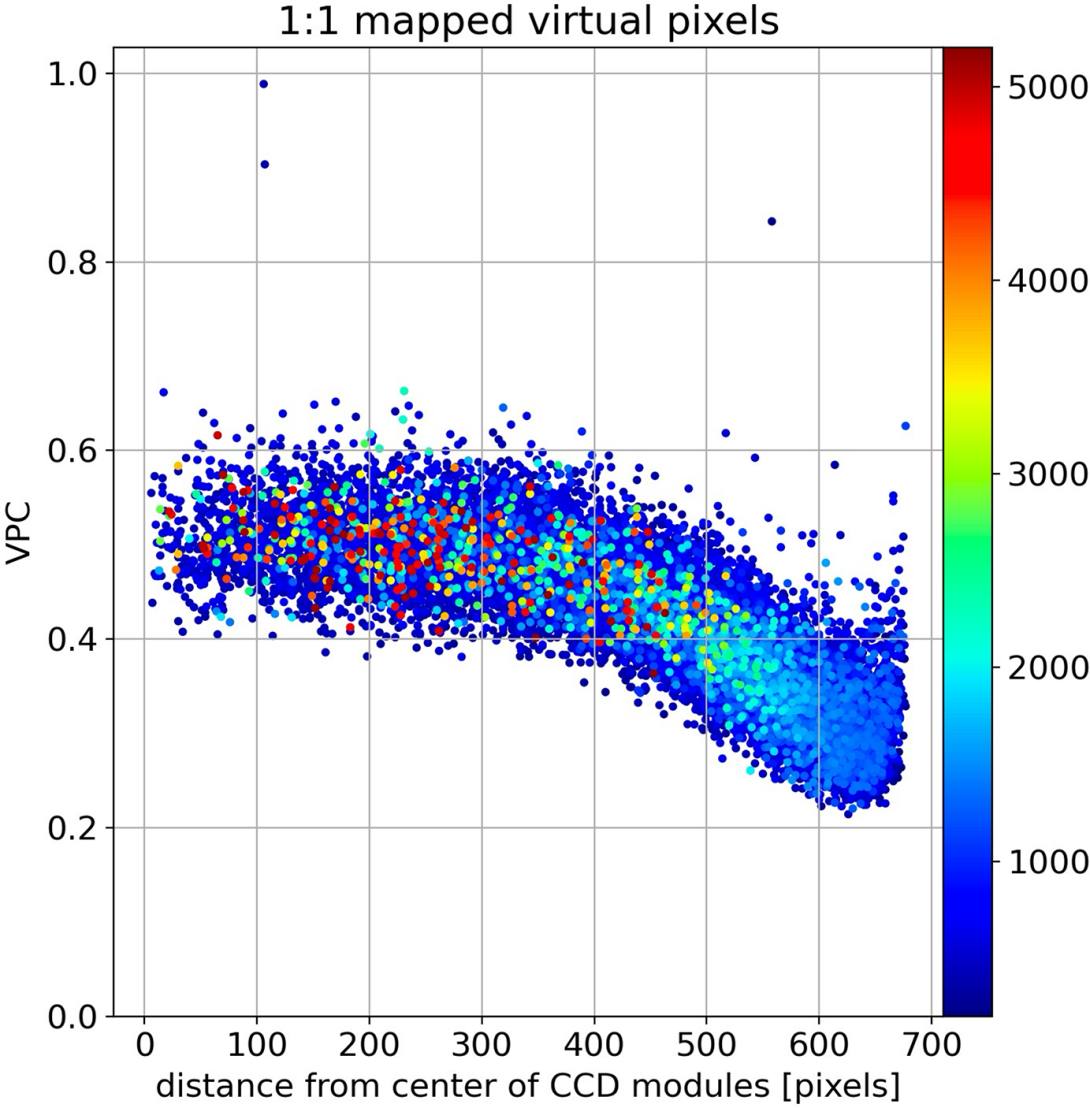
**VPC** for 22028 virtual pixels with 1:1 mapping to raw pixels. The color code corresponds to counts, which span from approximately 200 (blue) to 5000 (dark red).

According to photon counting statistics, **VPC** for virtual pixels mapped 1:1 from raw pixels should be equal to the square of the pixel DQE, which corresponds to the photon conversion gain in counts per photon. A chart of **VPC** for 1:1 mapped virtual pixels is shown in Fig. 10 ▸, which has a mean of 0.50 (DQE = 0.71) in the central region of the fiber tapers, *i.e.* within about 350 pixels from the fiber taper center, and drops to approximately 0.32 at a pixel distance that corresponds to the beamstop location, which is 593 pixels from center. The DQE estimated from this measurement is slightly smaller than the specified DQE of 0.8 for 12 keV photons. Color-coding **VPC** according to counts (which span from approximately 200 near the beam center to 5000 near the peak of the fused silica scattering ring) shows **VPC** is independent of counts, as expected. The systematic drop in **VPC** with increasing distance from the fiber taper center arises from an increasing probability of photon escape from the fiber core and into the cladding, which increases the pedestal contribution to the reported counts for each virtual pixel. Because the pedestal contribution represents an average over many neighboring pixels, it contributes negligibly to the measured variance. Hence, the systematic decrease in the variance with increasing distance from the fiber taper center is expected to correlate with the spot-to-pedestal ratio. Indeed, given this curve, we estimate the integrated intensity of the pedestal in the central region of the fiber taper to be approximately 10% of the total, which is down significantly from the 42% found experimentally near the beamstop. In retrospect, it would have been better to position the X-ray detector further off center so (X0, Y0) was positioned near the center of a fiber taper.

### 3.6. Detector linearity

The Rayonix response linearity was assessed using fused silica scattering datasets containing 512 images that were acquired at frame rates differing by a factor of two. Background-subtracted, zinger-free mean images were extracted from these datasets and denoted **FS1** and **FS2**, where **FS2** was acquired at twice the fame rate and has half the intensity. The standard deviation of the mean for **FS1**, which we denote **sigma**, is readily computed according to
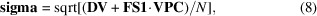
where **DV** corresponds to the detector readout variance, **VPC** corresponds to variance per count, and *N* is the number of images in the dataset.

**Figure 11:**
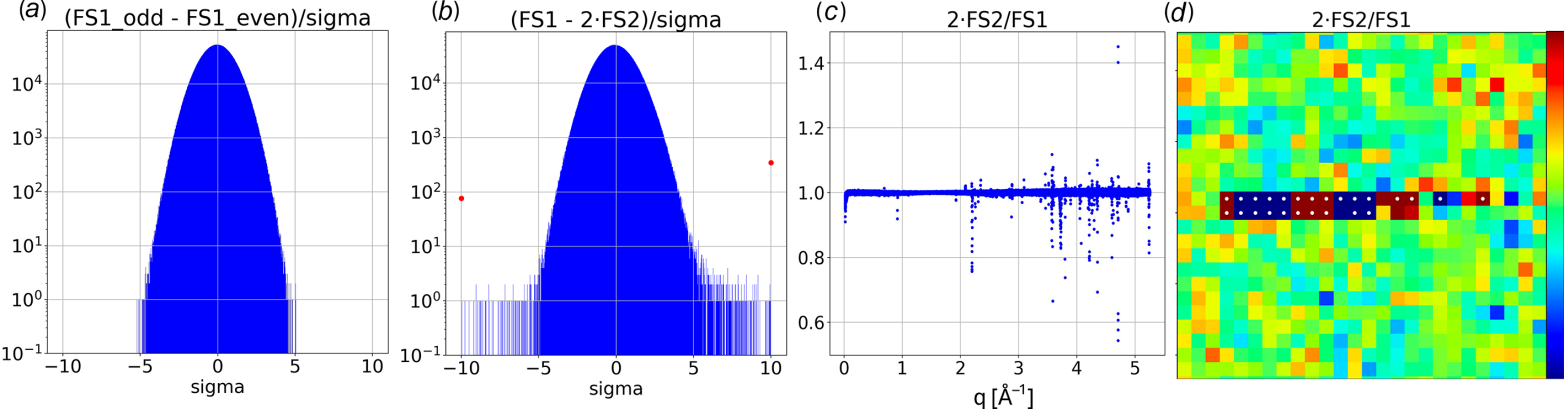
Rayonix detector statistics. (*a*) Histogram of relative error in sigma units when differencing zinger-free means computed from odd versus even frames in **FS1**. (*b*) Histogram of relative error in sigma units for the scaled difference **FS1** − 2·**FS2**. (*c*) Scatter chart of 2·**FS2**/**FS1** as a function of *q* identifies numerous outlier pixels. (*d*) Zoomed image of 2·**FS2**/**FS1** showing a cluster of outlier pixels found in the upper left corner of the detector. The image color code spans ±1% (dark red to dark blue).

To validate our estimate for **sigma**, the odd and even images from the **FS1** dataset were processed separately (256 images each) and their difference charted in Fig. 11 ▸(*a*) as a histogram in sigma units. As expected, the differences are normally distributed without outliers. With 14.6 million pixels represented in the histogram, the statistical probability distribution should be confined within ±5.27-**sigma**, as is the case.

Fig. 11 ▸(*b*) charts the **sigma**-normalized scaled difference (**FS1** − 2·**FS2**)/**sigma**. This distribution is similar to that in Fig. 11 ▸(*a*), which means most pixels scale linearly with X-ray exposure, but 845 pixels were found to differ by more than 6-**sigma**. The scaled ratio 2·**FS2**/**FS1** charted in Fig. 11 ▸(*c*) quantifies the relative magnitude of the error. Most of these outlier pixels are found in clusters, one of which is charted in Fig. 11 ▸(*d*) where the color code spans ±1% with the white dots identifying pixels that deviate from unity by more than 6-**sigma**.

The pixels flagged as outliers suffer gain error that is dependent on the detector frame rate. Since the count values reported by these outlier pixels are repeatable, a uniformity correction generated from experimental data at a standardized frame rate can correct for their gain error.

### 3.7. UC_FS_: uniformity correction from fused silica plate scattering

M0-scaling was used to isolate scattering from a 100 µm thick fused silica plate, from which a uniformity correction, **UC_FS_**, was generated. First, a dataset consisting of 512 scattering images recorded with the sample extracted from the X-ray beam was acquired and denoted **H**. Next, a dataset consisting of 512 scattering images with the fused silica plate inserted into the beam at normal incidence was acquired and denoted **FSH**, as it includes scattering contributions from both **FS** and **H**. Both datasets were acquired with a frame rate corresponding to 1.118 s per image. Note that the helium contribution to **FSH** is reduced relative to **H** due to the fused silica absorbance.

Using M0 scaling to isolate fused silica scattering **FS** from the **FSH** dataset is relatively straightforward,
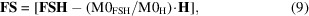
where M0 corresponds to the mean M0 for the dataset indicated by its subscript. Since scattering from a plate at normal incidence is circularly symmetric after polarization correction, we can use **FS/POL** to assess the detector response uniformity.

**Figure 12:**
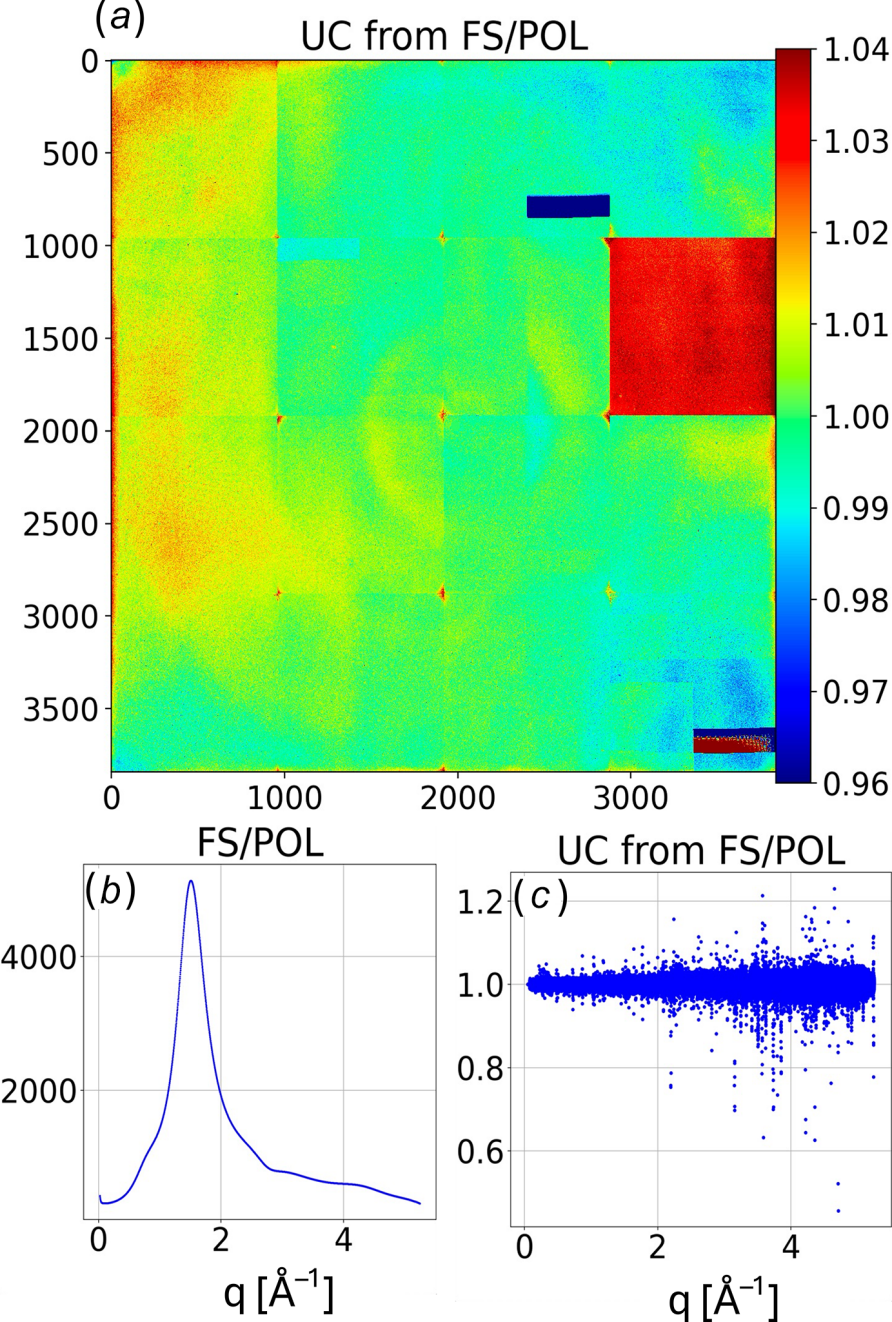
**FS/POL** scattering statistics. (*a*) Color-coded chart of **UC_FS_** uniformity correction with color scale spanning 0.96 (dark blue) to 1.04 (dark red). (*b*) Best-fit **FS**/**POL** scattering intensity is charted as a function of *q*. (*c*) Scatter chart of **UC_FS_** as a function of *q*. Perimeter pixels and pixels in defective regions of the detector were excluded.

First, the pixels in **FS/POL** were binned into *q*-bins spaced by 0.0025 Å^−1^. A linear least-squares fit of pixels in each bin, after rejection of 5σ statistical outliers, defines its *q*-dependent intensity, with **UC_FS_** defined as **FS/POL** divided by the linear least-squares fit for each bin. **UC_FS_** is charted as a color-coded image in Fig. 12 ▸(*a*) and is reasonably flat with minor scaling errors between most CCD modules except for one, which exhibits about 3.3% excess intensity. Pixels near the corners of the CCD modules tend to exhibit excess intensity due to scaling errors that presumably arise from the broad pedestal in the **PSF**. Hence, the ‘well behaved’ pixels used in the linear least-squares fits excluded pixels near the corners of modules, pixels along the perimeter of the detector, those affected by two ill-behaved ADCs, and those shadowed by the beamstop. Fig. 12 ▸(*b*) charts the best fit estimate of **FS/POL** for each *q*-bin. Note that the statistical precision of **UC_FS_** is limited by photon counting statistics and is poorest in the SAXS region where scattering from fused silica is weakest. The scatter chart of **UC_FS_** as a function of *q*, shown in Fig. 12 ▸(*c*), quantifies the magnitude of the scaling errors. Normalizing detector images acquired at the same frame rate by **UC_FS_** should correct these errors and put all pixels in the same *q*-bin on the same scale.

Due to spurious scattering near the beamstop, **UC_FS_** for pixels with *q* < 0.075 Å^−1^ were set to unity, which reverts this region to factory-calibrated scaling parameters. The spurious scattering likely arises from X-rays that scatter from edges on the upstream pinhole and reflect off inner surfaces of the brass collimator tip.

### 3.8. UC_GC_: uniformity correction from glassy carbon scattering

We acquired a **GC** dataset consisting of 512 scattering images from a 1 mm thick plate of glassy carbon. The attenuation length of glassy carbon at 12 keV, assuming a density of 1.5 g cm^−3^, is 5.2 mm. Hence, its estimated transmittance is 83%, which is comparable with a water-filled capillary. SAXS scattering from glassy carbon is very strong and required significant attenuation of the incident X-ray intensity to avoid saturating the detector. Indeed, its M0 barely registers above the background. Hence, the **GC** scattering images are free from both spurious and helium scattering. Thanks to its scattering strength and uniformity, glassy carbon has often been used as a standard reference material to characterize detector uniformity in SAXS setups (Allen *et al.*, 2017 ▸). Because its scattering intensity decreases rapidly with increasing *q*, dropping by an order of magnitude near 0.3 Å^−1^, it is useful only in the low *q* region. Nevertheless, a uniformity correction generated from glassy carbon can provide a high-precision substitute for **UC_FS_** in the SAXS region.

**Figure 13:**
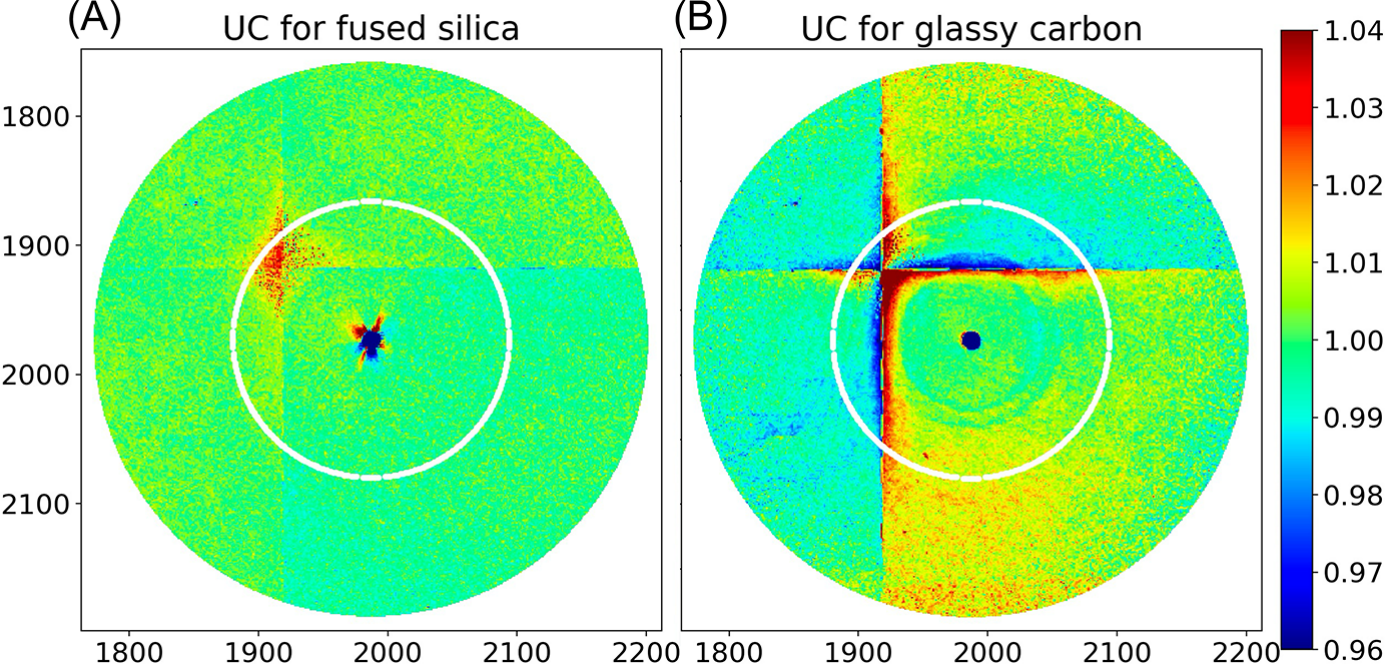
Uniformity correction for *q* < 0.6 Å^−1^. (*a*) Fused silica. (*b*) Glassy carbon. The white rings correspond to *q* = 0.3 Å^−1^, where the fused silica and glassy carbon scattering intensities are comparable.

To generate a uniformity correction from the **GC** dataset, we compute a background-subtracted, zinger-free average image, bin the data in that image, and then fit data in each bin using linear least squares. **UC_GC_**, which corresponds to the binned intensities divided by their fits in each bin, is shown in Fig. 13 ▸ alongside **UC_FS_** for *q* < 0.6 Å^−1^. The two uniformity corrections are similar, except for near the boundaries between CCD modules and near the beamstop. The relative scale of the four intersecting CCD modules appears to be slightly more consistent in **UC_FS_** than **UC_GC_**, but the differences are quite minor and can be ignored. The scattering intensity for fused silica over the charted range is weak and quite flat, but for glassy carbon is strong and rapidly decreasing, dropping below the scattering intensity of fused silica near *q* = 0.3 Å^−1^. Hence, the statistical precision for **UC_FS_** is better for *q* > 0.3 Å^−1^, whereas that for **UC_GC_** is better for *q* < 0.3 Å^−1^. As mentioned, spurious scattering contaminates the **FS** dataset, which leads to systematic scaling errors in **UC_FS_** near the beamstop. In contrast, **UC_GC_** is far more uniform near the beamstop and can be used as a substitute for that region in **UC_FS_**. As discussed, the broad pedestal contribution to the PSF leads to scaling errors near the boundaries between CCD modules. Those errors are far worse in **UC_GC_** than in **UC_FS_**. Indeed, the scaling errors are exacerbated by the strong gradient in scattering intensities across those boundaries.

### 3.9. Generating one-dimensional *I*(*q*) and *sigI*(*q*) from single images

Each recorded image, **I**, has a background offset, is contaminated with helium scattering and inelastic Compton scattering, suffers from scaling errors, and requires numerous corrections to convert **I** to **ICC**, a corrected image that has units of photons per solid angle and includes both **C**oherent (elastic) and **C**ompton (inelastic) scattering. First, we use M0 scaling to remove the helium contribution to the background-subtracted scattering and then apply all relevant scaling corrections according to
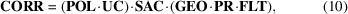

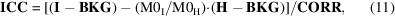

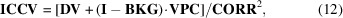
where **CORR** is a composite correction factor whose first two terms in parentheses depend on both ϕ and ψ. **SAC** corresponds to the sample absorbance correction, which for a plate scatterer is characterized by **PT** and depends on only ψ, but for a capillary (to be discussed elsewhere), depends on both ϕ and ψ. The last three terms in parentheses depend on ϕ only. **ICCV** is the variance of **ICC** as predicted by **I**, **BKG**, **DV**, **VPC** and **CORR**. Next, we bin **ICC** according to *q*, which spans from 0.02 to over 5.2 Å^−1^ in steps of 0.0025, and find the median value in each bin, **ICCM**. We flag zingers according to
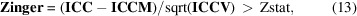
where Zstat corresponds to the *Z* statistic for which normally distributed data would generate one false positive per image. For 14.6 million pixels, Zstat = 5.27. For computational convenience, pixels flagged as zingers are replaced with their corresponding **ICCM** and a sigma-weighted linear least squares fit is performed on the *I*_*qi*_ contents in each *q* bin. The least-squares fit evaluated at *q* is denoted *I*(*q*) and the fit residual standard deviation of the mean is denoted *sigI*(*q*) according to
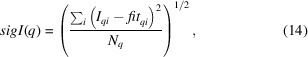
where *N*_*q*_ is the number of pixels *i* assigned to bin *q*.

**Figure 14:**
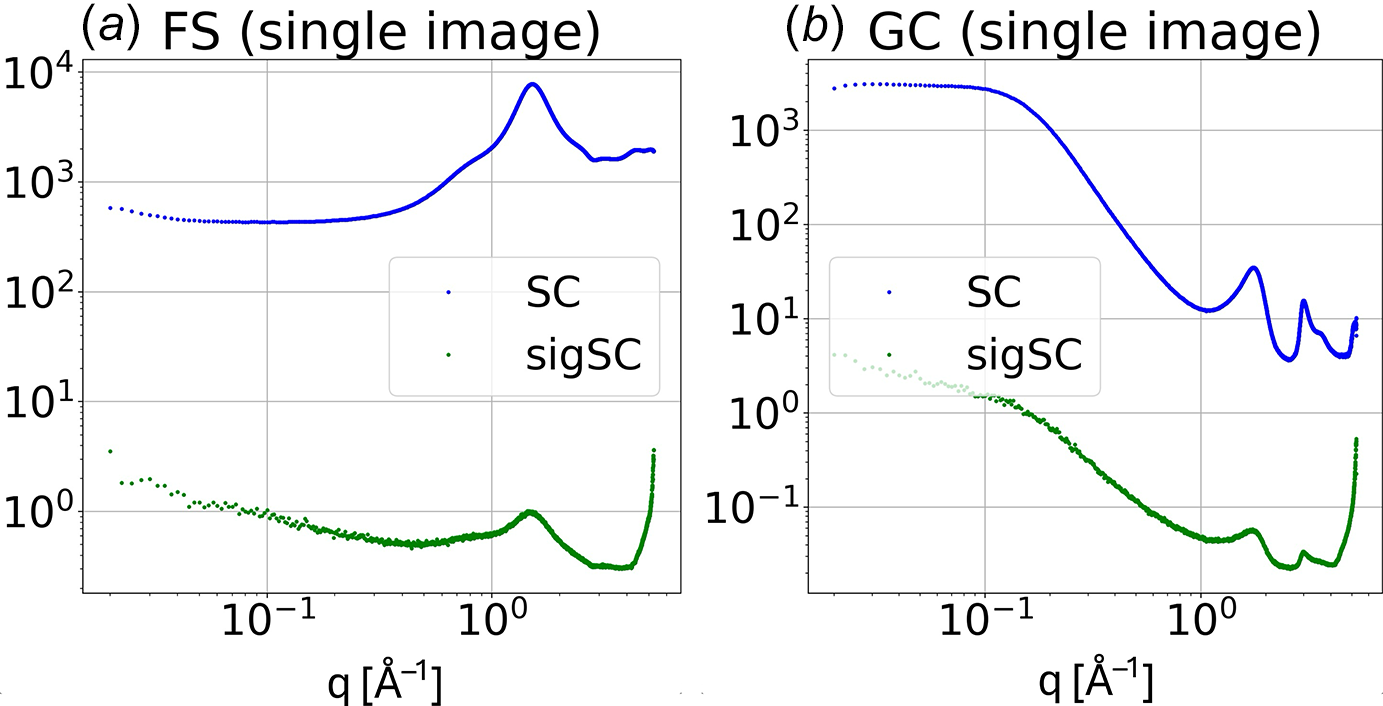
Log–log charts of *I*(*q*) and *sigI*(*q*) for single images acquired in just over 1 s. (*a*) Fused silica. (*b*) Glassy carbon.

*I*(*q*) and *sigI*(*q*) are charted for single images of fused silica and glassy carbon scattering in Fig. 14 ▸. The **UC** used for the fused silica dataset was generated from that dataset, *i.e.***UC_FS_**, which puts all pixels at the same *q* on the same scale. The **UC** used for the glassy carbon dataset up to 0.6 Å^−1^ was derived from that dataset, *i.e.***UC_GC_**, but beyond that threshold, where the glassy carbon scattering intensity becomes very weak, **UC_FS_** was used (see Fig. 13 ▸ for comparison of **UC_FS_** and **UC_GC_** below 0.6 Å^−1^). This hybrid approach ensured accurate scaling over the full range of *q*. Each scattering curve represents the average of 160 X-ray shots, each consisting of a 500 ns duration pulse train, and was acquired in just over 1 s. The log–log charts emphasize the SAXS region and demonstrate the high signal-to-noise (S/N) achievable with a pulsed X-ray source. Thanks to the large number of pixels in the WAXS region, the maximum S/N achieved in the fused silica scattering curve approaches 10000:1, which is crucial to reliably extract WAXS scattering signatures from biomolecules whose highest concentration is typically less than 20 mg ml^−1^, or approximately 1.4% by volume. Note that the S/N rapidly degrades at high *q* due to the decreasing number of pixels available in those bins. The time resolution in this mode of operation is limited by the duration of the superbunch to 500 ns. When operating in single bunch mode, the time resolution can be improved to 120 ps, but with 5.4 times fewer X-ray photons per shot the S/N would be reduced by a factor of 2.3. When sources of systematic error are properly managed, signal averaging of multiple images can be used to further improve the S/N according to the square root of the number of images averaged.

### 3.10. PSF-induced scaling error near beamstop

**Figure 15:**
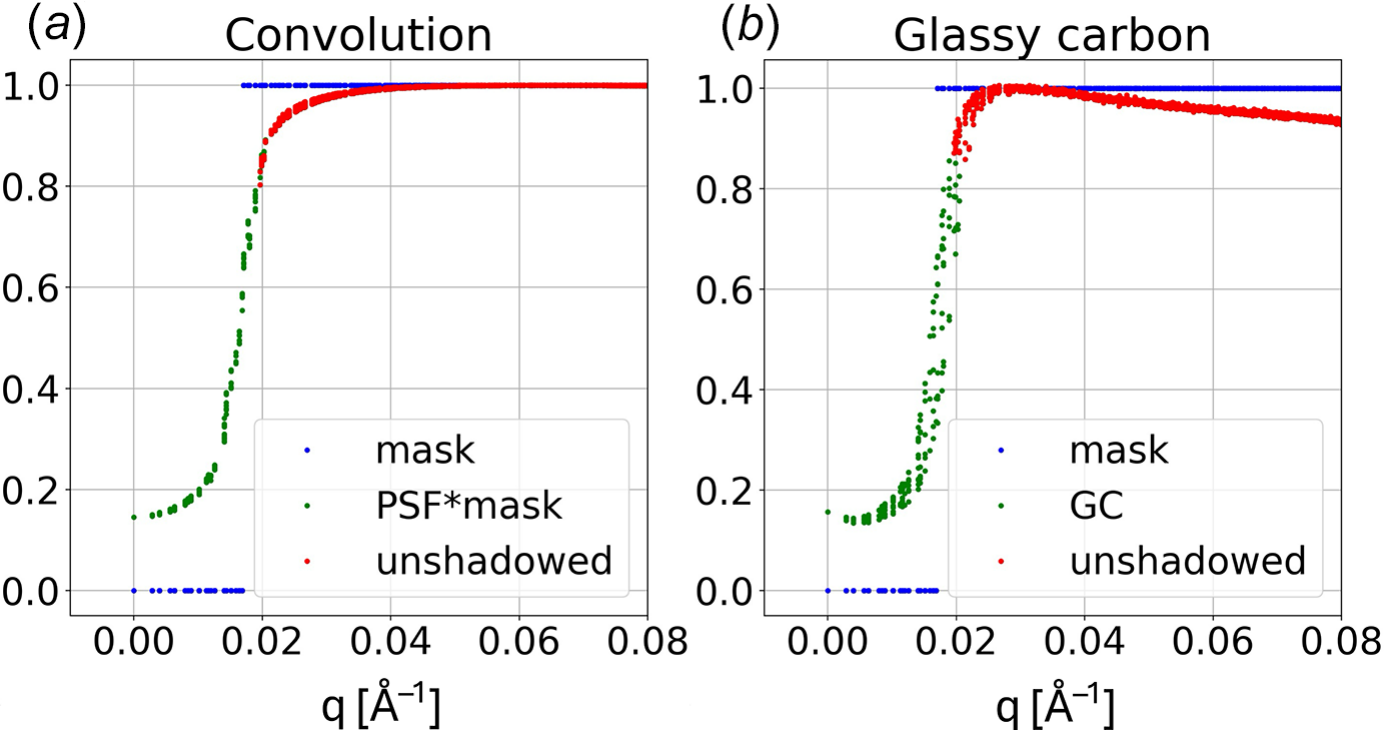
Scattering near the beamstop. (*a*) Theoretical scattering intensity distribution, as determined by convolution of **PSF** with a **mask** that simulates the hard edge of the beamstop. (*b*) Normalized glassy carbon scattering near the beamstop. The red dots in (*a*) and (*b*) correspond to pixels outside the beamstop shadow.

The glassy carbon scattering in Fig. 14 ▸(*b*) exhibits a decrease in scattering intensity near the beamstop, which is due to scaling errors arising from the broad pedestal contribution to the **PSF**. Theoretically, scattering images recorded by the Rayonix detector correspond to the convolution of its **PSF** with the true scattering intensity distribution. For example, if we assume the scattering intensity is constant at low *q* but blocked by the beamstop, the expected intensity distribution can be simulated with a 2D **mask** that is set to zero inside the hard edge of the beamstop, which for this setup corresponds to six pixels from center. Note that this mask differs from the beamstop mask, which is nominally seven pixels from center and includes partially shadowed pixels. The convolution of **PSF** with **mask**, *i.e.*PSF*mask, is shown in Fig. 15 ▸(*a*). It is because of the broad pedestal in the **PSF** that the scattering intensity does not go to zero under the beamstop. The scattering offsets under the beamstop and the slopes of the scattering intensity near the beamstop edges are very similar for the convolution and the experimental glassy carbon data shown in Fig. 15 ▸(*b*), which validates our measurement of the **PSF**. The red dots in Fig. 15 ▸ corresponds to pixels that are not shadowed and can be used to characterize SAXS scattering. Their observed intensity in Fig. 15 ▸(*a*) begins to diverge from the ‘true’ intensity around *q* = 0.04 Å^−1^ and deviates by up to 20% at the beamstop edge. This factor agrees with the observation that slightly more than 40% of the PSF integrated intensity is found in the broad pedestal, and, for pixels next to the beamstop shadow, nearly half of that pedestal is shadowed by the beamstop, thereby reducing the number of photons detected by approximately half of 40%, or 20%. This result helps to explain why the glassy carbon scattering intensity in Fig. 15 ▸(*b*) decreases as it approaches the beamstop edge. Since scattering data acquired near the beamstop edge is systematically suppressed in a predictable way, it can be rescaled to recover its true intensity distribution. With this correction, reliable scattering amplitudes can be acquired down to *q* = 0.02 Å^−1^. When not properly corrected, this distortion can depress the experimental determination of both I0 (the forward scattering intensity at *q* = 0) and Rg (the radius of gyration). Since the magnitude of this correction depends on the intensity gradient near the beamstop, an iterative procedure is needed to properly correct for this scaling error, as will be discussed elsewhere.

### 3.11. Putting *I*(*q*) on an absolute, atomic scale

In the absence of molecular structure, the expected scattering intensity *I*(*q*) for a molecule is proportional to 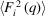 + 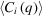, where 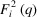 represents the square of the atomic form factor, *C*_*i*_(*q*) corresponds to incoherent Compton scattering, and the brackets indicate the stoichiometrically weighted average over *i* elements with the sum of the weights normalized to unity. Here, we generate *F*_*i*_(*q*) and *C*_*i*_(*q*) by cubic spline interpolation between tabulated values reported by Hubbell *et al.* (1975 ▸). Note that *F*_*i*_(*q*) at *q* = 0 is equal to the atomic number of element *i* (*Z*_*i*_) and decays to zero at high *q*. In contrast, *C*_*i*_(*q*) is zero at *q* = 0 but increases to *Z*_*i*_ at high *q*, surpassing 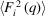 near 9.27 Å^−1^ in fused silica, 5.47 Å^−1^ in water, and 4.79 Å^−1^ in glassy carbon. Hence, one would expect Compton scattering for biomolecules, which consist primarily of C, N and O, to be comparable in amplitude to coherent scattering near 5 Å^−1^. Studies that focus on the SAXS region can ignore Compton scattering, but it cannot be ignored here. Note that the incoherent scattering function in Hubbell *et al.* is denoted *S*, but here we denote it *C* for Compton scattering, as *S* is reserved for the structure factor, which is discussed below.

**Figure 16:**
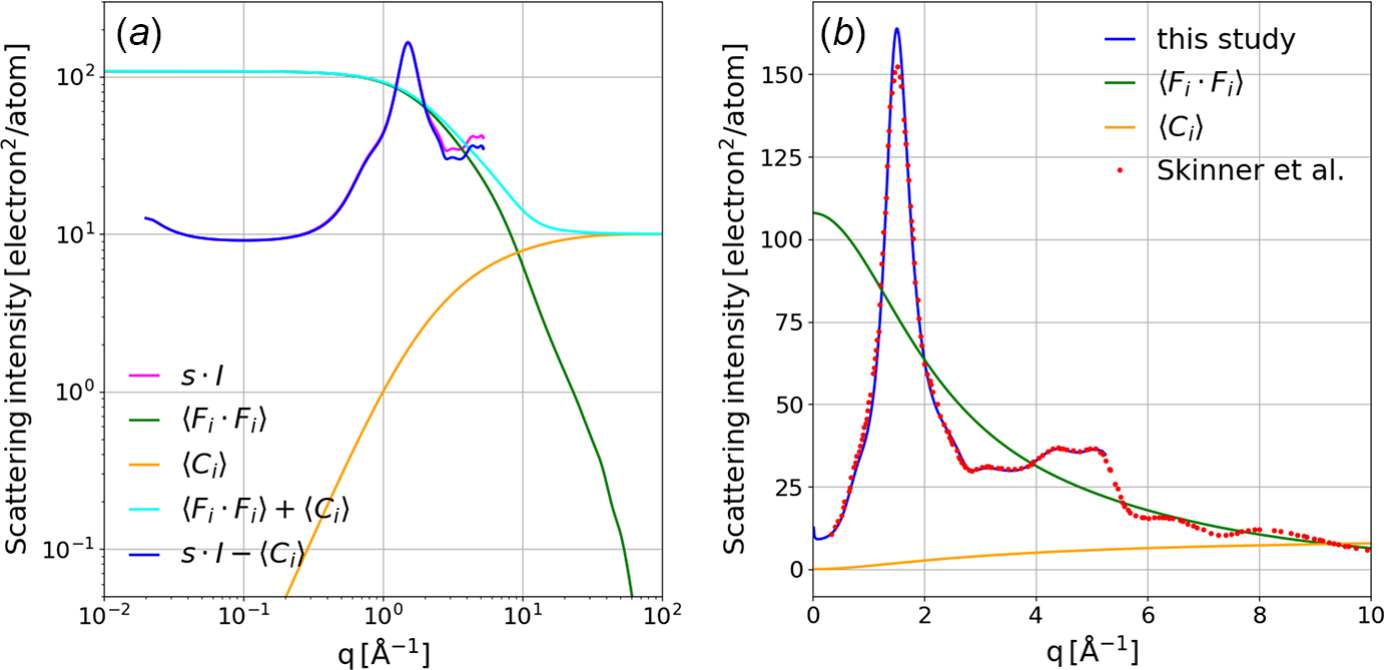
Fused silica scattering. (*a*) Log–log chart of theoretical (atomic) and measured scattering for fused silica. The quantities in brackets represent stoichiometrically weighted means. The magenta curve, *sI*, has been scaled (see text) to put *I*(*q*) on the same absolute scale as the cyan curve, 〈*F*_*i*_·*F*_*i*_〉 + 〈*C*_*i*_〉. The blue curve corresponds to *I*_*c*_ (= *sI* − 〈*C*_*i*_〉), the Compton-corrected coherent scattering. (*b*) *I*_*c*_ overlaid with data extracted from Fig. 1 of Skinner *et al.* (2012 ▸).

The theoretical elastic 〈*F*_*i*_(*q*)^2^〉 and inelastic 〈*C*_*i*_(*q*)〉 atomic scattering curves for fused silica are shown in Fig. 16 ▸ along with their sum and *I*(*q*) times a scale factor, *s*, that puts the experimentally determined scattering curve on the same scale. How can we reliably determine *s*?

Fused silica is a glass comprising SiO_2_ and lacks long range order; however, it has local structure that arises from chemical bonding between neighboring silicon and oxygen atoms. Scattering from neighboring atoms can interfere both constructively and destructively, which leads to modulation in *I*(*q*). These interference effects are described by its structure factor, *S*(*q*), according to
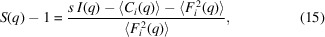
where *s* is the scale factor that puts *I*(*q*) on the same scale as 〈*C*_*i*_(*q*)〉, after which Compton scattering can be properly subtracted. In this equation, 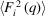 represents the expected elastic scattering curve in the absence of local structure, which is subtracted from the scaled, Compton-corrected scattering curve and the resulting difference is normalized by 〈*F*_*i*_(*q*)^2^〉, the square of the mean form factor. Given *S*(*q*), the molecular pair distribution function *p*(*r*) can be determined.

Since local molecular structure causes the measured scattering intensity to porpoize about 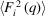 + 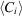 in the WAXS region, the ratio of integrated theoretical and experimental scattering curves over a judiciously selected range of *q* should provide a reasonably accurate estimate for *s*,
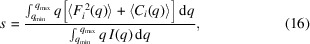
where the scattering amplitudes are weighted by *q* in recognition of the fact that the theoretical and experimental scattering curves are one-dimensional representations of two-dimensional scattering images, with the number of pixels used to quantify *I*(*q*) being proportional to *q*. What is meant by ‘judiciously selected range of *q*’? The average of sinusoidal modulation from its midpoint over a full period is zero. Hence, if we assign *q*_min_ and *q*_max_ to the midpoints of the rising edges for the first and last well resolved peaks in *I*(*q*), the integral over that range of *q* will be minimally biased by its modulation. For fused silica, the integral was computed from 1.25 to 4 Å^−1^, as shown in Fig. 16 ▸(*a*). In Fig. 16 ▸(*b*), our Compton-corrected scattering curve *I*_*c*_(*q*) = *sI*(*q*) − 〈*C*_*i*_(*q*)〉 is overlaid with fused silica scattering data extracted from Fig. 1 of Skinner *et al.* (2012 ▸), which was acquired with 100 keV X-rays and a 1 mm thick fused silica plate. The quantitative agreement beyond 2 Å^−1^ validates not only the approach used to scale our fused silica scattering data but also the accuracy of the Rayonix uniformity correction used when processing these data. The differences observed at lower *q* are real and likely arise from samples with different thermal history, which can affect the sample density as well as the density fluctuations frozen into the sample when cooled below its glass transition temperature.

### 3.12. Real space resolution

The high-resolution hard limit for our scattering data is approximately 2π/5.2 Å^−1^ = 1.2 Å, which is modestly greater than the shortest bond distance known for heavy atoms, *i.e.* 1.098 Å for the di­nitro­gen triple bond, N≡N. X-ray scattering studies of fused silica at much higher spatial resolution demonstrated that the high *q* scattering pattern can be quantitatively reproduced with three interatomic distances: Si—O (1.616 Å), O—O (2.637 Å) and Si—Si (3.099 Å) (Narten, 1972 ▸). Our 1.2 Å resolution limit is insufficient to resolve O—O and Si—Si peaks in the pair distribution function *p*(*r*) generated from our data due to their close proximity. Nevertheless, since these distances are known, their contribution to the WAXS scattering at high *q* is predictable. Indeed, when studying biomolecules such as proteins, RNAs and DNAs, the primary structure is known and scattering beyond our hard limit can be predicted with relatively good accuracy. Scattering recorded within our high-resolution limit, if acquired with sufficiently high S/N, should provide a ‘fingerprint’ that is sensitive to secondary, tertiary and quaternary structure, which from a biophysical point of view is much more interesting. For example, Köfinger & Hummer (2013 ▸) calculated *p*(*r*) directly from biomolecule molecular dynamic simulations and compared it with *p*(*r*) calculated indirectly via Fourier transformation of *I*(*q*) generated from the same simulations but after truncation at *q*_max_. They concluded that most of the features in *p*(*r*) are well reproduced with *q*_max_ = 3 Å^−1^, which is well within our experimental limit of 5.2 Å^−1^.

### 3.13. SAXS resolution

**Figure 17:**
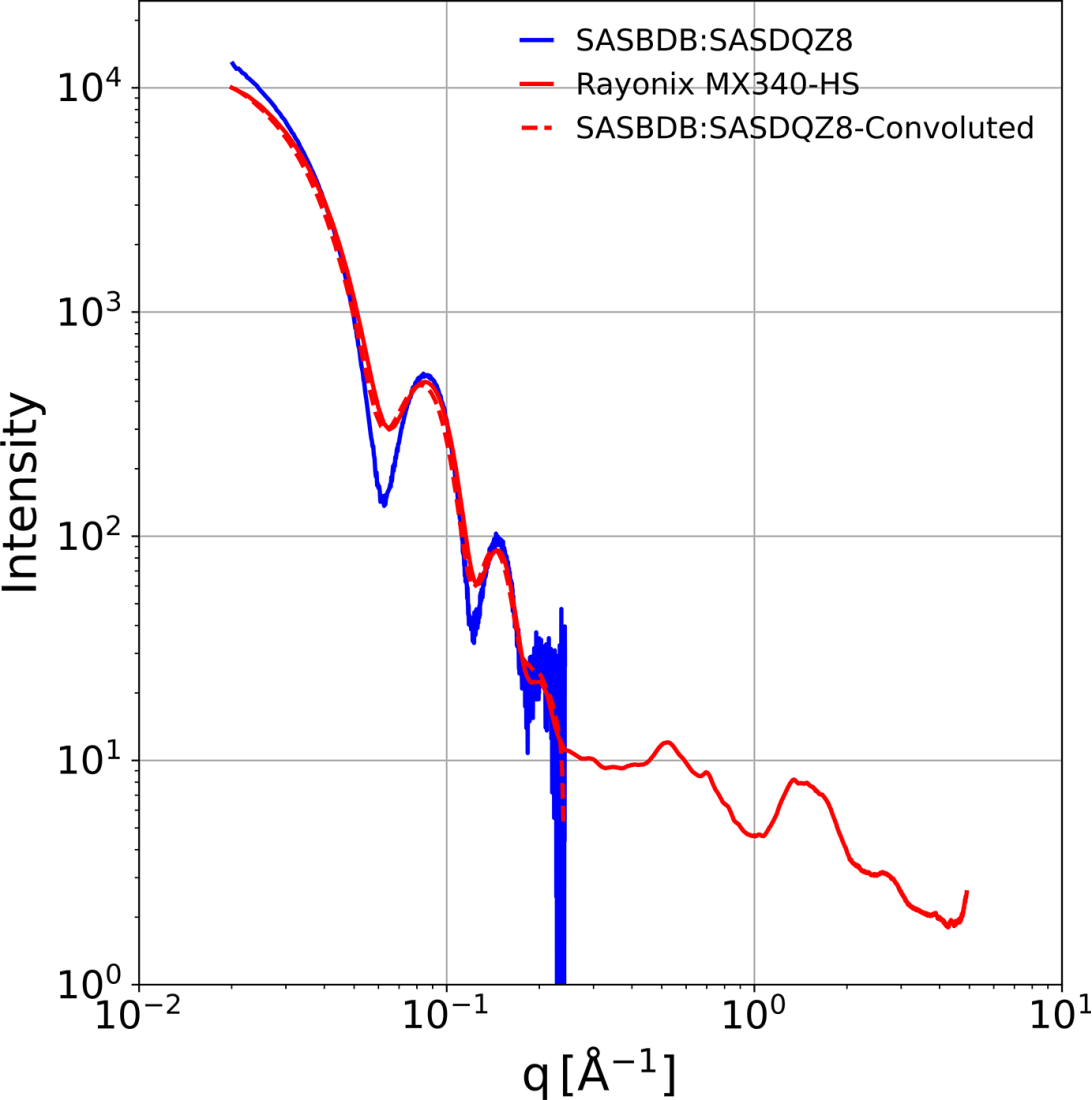
Apoferritin scattering. SAXS scattering (SASBDB code: SASDQZ8) recorded by XFEL (blue) (Blanchet *et al.*, 2023 ▸), SAXS/WAXS scattering recorded with the Rayonix detector on the BioCARS time-resolved beamline (red), and convolution of our point-spread function with the XFEL scattering curve (red dashed line).

The X-ray energy, detector size, sample-to-detector distance, beamstop size and beamstop location define the range of *q* that can be accessed with a single X-ray detector, and its pixel size limits its achievable SAXS resolution. With our geometry, the range of *q* is 0.02 to 5.2 Å^−1^, and the diagonal of the 89 µm square Rayonix pixels translates to a worst case on-axis resolution limit of 0.004 Å^−1^. When converting zinger-free, normalized 2D scattering images to one-dimensional *Ic*(*q*) scattering curves, we sort the scattering amplitudes according to *q*_i_, bin them in 0.0025 Å^−1^ wide *q*-bins, perform a weighted linear least-squares fit of the data in each *q*-bin, and solve for the best-fit amplitude at the corresponding value of *q*. Hence, the SAXS resolution for *Ic*(*q*) is fundamentally limited not by bin size but by pixel size. Worse, the resolution actually achieved is defined by a convolution of the pixel-limited resolution with the detector **PSF**. For large biomolecules such as apoferritin, our limited pixel resolution in the SAXS region and the detector **PSF** reduces the modulation amplitude of features relative to those observed in more traditional SAXS setups, where the sample-to-detector distance is much larger and the detector **PSF** is typically confined to one pixel. Nevertheless, since we know our detector **PSF**, we can account for its effect on the scattering pattern. For example, we overlay in Fig. 17 ▸X-ray scattering of apoferritin (Sigma, A3660) recorded with our setup (details to be published elsewhere) and a scattering curve (SASBDB code: SASDQZ8) recorded by X-ray free-electron laser (XFEL) (Blanchet *et al.*, 2023 ▸), whose low *q* cutoff is 0.02 Å^−1^, the same as our setup. Also included in this chart is a convolution of the XFEL curve with our **PSF**, which reproduces our Rayonix data from 0.02 to approximately 0.2 Å^−1^ with high fidelity. Hence, it should prove possible to deconvolve our high S/N SAXS data, subject to appropriate constraints, to recover accurate, corrected SAXS curves. Note that the S/N of the XFEL data become poor near 0.2 Å^−1^, whereas our scattering data unveil modulation of the scattering intensity with high S/N out to at least 4 Å^−1^. Since the **PSF**-induced smearing of the SAXS scattering curve at low *q* is largely confined to *q* < 0.04, for biomolecules that are small compared with apoferritin, this region can be masked without compromising Guinier analysis of the scattering curve.

### 3.14. **PSF** implications for crystallography studies

It is worth noting that the Rayonix-generated uniformity correction is derived from flood-field illumination at a distance, which means uniformity is achieved independent of the **PSF** spot-to-pedestal ratio. In crystallography, integrated spot intensities are determined after background subtraction. Since the spot-to-pedestal integrated intensity ratio differs according to distance from the fiber taper center, one would expect the measured spot intensities to systematically decrease relative to their true value with increasing distance from the fiber taper center. In principle, a scale factor parameterized in terms of this distance could be applied to crystallography data, which should improve the precision of integrated spot intensities and lower their *R*-factor.

## 4. Summary

We described an X-ray scattering setup capable of acquiring high S/N scattering images with short duration X-ray pulses over a broad range of *q* spanning 0.02 to over 5.2 Å^−1^ with a single X-ray detector. X-ray leakage through small diameter, multi-composition, partially transmissive beamstop is used to not only maintain precise beam alignment over time but also to provide an internal standard for quantifying X-ray dosage for each image, which is useful to properly scale images prior to differencing. A detailed statistical analysis of dark images and fused silica scattering images led us to introduce a **VPC** statistic, which facilitates flagging of zingers and generation of statistically weighted averages when converting 2D images to one-dimensional scattering curves. The X-ray detector **PSF** has a weak, broad pedestal that leads to significant scaling errors near boundaries between CCD modules and near the edge of the beamstop; however, these scaling errors can be largely corrected by an experimentally determined uniformity correction according to methods described herein. Finally, we report X-ray scattering data from a solution of apoferritin and show how the **PSF** affects the SAXS resolution in a predictable fashion. Though evident in apoferritin scattering, the effect is minimal with moderately sized biomolecules. Finally, the open sample environment in this setup enables acquisition of scattering data from samples perturbed by a variety of methods including laser-based pump–probe or stopped flow mixing and should prove very useful in studies that aim to probe biomolecule structure and dynamics on fast time scales. Indeed, our ability to track oscillations in the X-ray scattering curve over a very wide dynamic range of *q* with high S/N should lead to far deeper structural insights than can be gleaned from SAXS data alone. However, the analysis required to gain these insights is beyond the scope of this manuscript and will be discussed elsewhere.

Note that fiber-taper-coupled detectors share many of the limitations addressed here, including susceptibility to zingers, position-dependent point-spread functions, and scaling errors arising from phosphor illumination through a fiber taper. Accordingly, the approaches developed in this work to improve the accuracy of the Rayonix detector should be broadly applicable to other fiber-taper-coupled detectors. X-ray detectors based on mosaics of direct-detection sensors are far less vulnerable to zingers, and their point-spread functions do not exhibit a broad pedestal. However, such detectors are typically smaller, requiring shorter sample-to-detector distances to achieve comparable angular coverage, and do not generate gap-free images. Nevertheless, for high-precision scattering measurements, direct detectors can benefit from experimentally derived uniformity corrections obtained at the appropriate sample-to-detector distance, following the general approaches described here.

## Figures and Tables

**Table 1:** Properties of materials used in the partially transmissive beamstop The attenuation length corresponds to its 1/*e* transmission. Ablation occurs when heating the surface exposed material to its boiling point.

Property	Aluminium	Selenium	Tantalum
Boiling point (°C)	2470	684.8	5457
Melting point (°C)	660.3	217	3017
Heat capacity (J g^−1^ K^−1^)	0.90	0.321	0.14
Density (g cm^−3^)†	2.70	4.5	16.65
Heat capacity (J cm^−3^ K^−1^)	2.43	1.44	2.33
12 keV attenuation length (µm)†	257	81.0	2.66
24 keV attenuation length (µm)†	1988	75.1	16.0
Path length (µm)	3200	500	85 (wall thickness)

† https://henke.lbl.gov/optical_constants/atten2.html.

## Data Availability

The data that support the findings of this study are available from the corresponding author upon reasonable requests.

## References

[bb1] Alkire, R. W., Rotella, F. J., Duke, N. E. C., Otwinowski, Z. & Borek, D. (2016). *J. Appl. Cryst.* **49**, 415–425. 10.1107/S1600576716000431 PMC4815871 27047303

[bb2] Allen, A. J., Zhang, F., Kline, R. J., Guthrie, W. F. & Ilavsky, J. (2017). *J. Appl. Cryst.* **50**, 462–474. 10.1107/S1600576717001972 PMC5377342 28381972

[bb3] Barna, S. L., Tate, M. W., Gruner, S. M. & Eikenberry, E. F. (1999). *Rev. Sci. Instrum.* **70**, 2927–2934.

[bb4] Blanchet, C. E., Round, A., Mertens, H. D. T., Ayyer, K., Graewert, M., Awel, S., Franke, D., Dörner, K., Bajt, S., Bean, R., Custódio, T. F., de Wijn, R., Juncheng, E., Henkel, A., Gruzinov, A., Jeffries, C. M., Kim, Y., Kirkwood, H., Kloos, M., Knoška, J., Koliyadu, J., Letrun, R., Löw, C., Makroczyova, J., Mall, A., Meijers, R., Pena Murillo, G. E., Oberthür, D., Round, E., Seuring, C., Sikorski, M., Vagovic, P., Valerio, J., Wollweber, T., Zhuang, Y., Schulz, J., Haas, H., Chapman, H. N., Mancuso, A. P. & Svergun, D. (2023). *Commun. Biol.* **6**, 1057. 10.1038/s42003-023-05416-7 PMC10585004 37853181

[bb5] Bösecke, P. & Diat, O. (1997). *J. Appl. Cryst.* **30**, 867–871.

[bb6] Cho, H. S., Dashdorj, N., Schotte, F., Graber, T., Henning, R. & Anfinrud, P. (2010). *Proc. Natl Acad. Sci. USA* **107**, 7281–7286. 10.1073/pnas.1002951107 PMC2867760 20406909

[bb7] Cho, H. S., Schotte, F., Dashdorj, N., Kyndt, J., Henning, R. & Anfinrud, P. A. (2016). *J. Am. Chem. Soc.* **138**, 8815–8823. 10.1021/jacs.6b03565 PMC5336379 27305463

[bb8] Cho, H. S., Schotte, F., Stadnytskyi, V. & Anfinrud, P. (2021). *Curr. Opin. Struct. Biol.* **70**, 99–107. 10.1016/j.sbi.2021.05.002 PMC8530917 34175665

[bb9] Cho, H. S., Schotte, F., Stadnytskyi, V., DiChiara, A., Henning, R. & Anfinrud, P. (2018). *J. Phys. Chem. B* **122**, 11488–11496. 10.1021/acs.jpcb.8b07414 PMC6580858 30285440

[bb10] Coleman, C. I. (1985). *Photo-Electronic Image Devices, Proceedings of the Eight Symposium*, pp. 649–661. Elsevier.

[bb11] Graber, T., Anderson, S., Brewer, H., Chen, Y.-S., Cho, H. S., Dashdorj, N., Henning, R. W., Kosheleva, I., Macha, G., Meron, M., Pahl, R., Ren, Z., Ruan, S., Schotte, F., Šrajer, V., Viccaro, P. J., Westferro, F., Anfinrud, P. & Moffat, K. (2011). *J. Synchrotron Rad.* **18**, 658–670. 10.1107/S0909049511009423 PMC3121234 21685684

[bb12] Gruner, S. M., Barna, S. L., Wall, M. E., Tate, M. W. & Eikenberry, E. F. (1993). *Proc. SPIE* **2009**, 98–108.

[bb13] Henning, R. W., Kosheleva, I., Šrajer, V., Kim, I.-S., Zoellner, E. & Ranganathan, R. (2024). *Struct. Dyn.* **11**, 014301. 10.1063/4.0000238 PMC10834067 38304444

[bb14] Holton, J. M., Nielsen, C. & Frankel, K. A. (2012). *J. Synchrotron Rad.* **19**, 1006–1011. 10.1107/S0909049512035571 PMC3480276 23093762

[bb15] Hubbell, J. H., Veigele, W. J., Briggs, E. A., Brown, R. T., Cromer, D. T. & Howerton, R. J. (1975). *J. Phys. Chem. Ref. Data* **4**, 471–538.

[bb16] Jeffries, C. M., Ilavsky, J., Martel, A., Hinrichs, S., Meyer, A., Pedersen, J. S., Sokolova, A. V. & Svergun, D. I. (2021). *Nat. Rev. Methods Primers* **1**, 70.

[bb17] Kirby, N. M. & Cowieson, N. P. (2014). *Curr. Opin. Struct. Biol.* **28**, 41–46. 10.1016/j.sbi.2014.07.007 25108308

[bb18] Koch, M. H. J. (2010). *J. Phys. Conf. Ser.* **247**, 012001.

[bb19] Köfinger, J. & Hummer, G. (2013). *Phys. Rev. E* **87**, 052712. 10.1103/PhysRevE.87.052712 23767571

[bb20] Krosigk, G. V., Cunis, S., Gehrke, R. & Kranold, R. (2001). *Nucl. Instrum. Methods Phys. Res. A* **467–468**, 1088–1091.

[bb21] Lyngsø, J. & Pedersen, J. S. (2021). *J. Appl. Cryst.* **54**, 295–305.

[bb22] Makowski, L. (2010). *J. Struct. Funct. Genomics* **11**, 9–19. 10.1007/s10969-009-9075-x PMC3057577 20049539

[bb23] Narten, A. H. (1972). *J. Chem. Phys.* **56**, 1905–1909.

[bb24] Pauw, B. R. (2014). *J. Phys. Condens. Matter* **26**, 239501. 10.1088/0953-8984/26/23/239501 24977264

[bb25] Skinner, L. B., Benmore, C. J. & Parise, J. B. (2012). *Nucl. Instrum. Methods Phys. Res. A* **662**, 61–70.

[bb26] Svergun, D. I. & Koch, M. H. J. (2002). *Curr. Opin. Struct. Biol.* **12**, 654–660. 10.1016/s0959-440x(02)00363-9 12464319

[bb27] Uesugi, K., Hoshino, M. & Yagi, N. (2011). *J. Synchrotron Rad.* **18**, 217–223. 10.1107/S0909049510044523 PMC3042329 21335908

[bb28] Wu, H. & Li, Z. (2024). *J. Synchrotron Rad.* **31**, 1197–1208. 10.1107/S1600577524007392 PMC11371043 39182204

